# Prionoids in amyotrophic lateral sclerosis

**DOI:** 10.1093/braincomms/fcac145

**Published:** 2022-06-09

**Authors:** Philippe Gosset, William Camu, Cedric Raoul, Alexandre Mezghrani

**Affiliations:** INM, Univ Montpellier, INSERM, CNRS, Montpellier 34095, France; INM, Univ Montpellier, INSERM, CNRS, Montpellier 34095, France; ALS Center, Univ Montpellier, CHU Montpellier, Montpellier, France; INM, Univ Montpellier, INSERM, CNRS, Montpellier 34095, France; Laboratory of Neurobiology, Kazan Federal University, Kazan, Russia; INM, Univ Montpellier, INSERM, CNRS, Montpellier 34095, France

**Keywords:** amyotrophic lateral sclerosis, prion-like, protein aggregation, physiopathology, therapy

## Abstract

Amyotrophic lateral sclerosis (ALS) is the third most frequent neurodegenerative disease after Alzheimer’s and Parkinson’s disease. ALS is characterized by the selective and progressive loss of motoneurons in the spinal cord, brainstem and cerebral cortex. Clinical manifestations typically occur in midlife and start with focal muscle weakness, followed by the rapid and progressive wasting of muscles and subsequent paralysis. As with other neurodegenerative diseases, the condition typically begins at an initial point and then spreads along neuroanatomical tracts. This feature of disease progression suggests the spreading of prion-like proteins called prionoids in the affected tissues, which is similar to the spread of prion observed in Creutzfeldt-Jakob disease. Intensive research over the last decade has proposed the ALS-causing gene products Cu/Zn superoxide dismutase 1, TAR DNA-binding protein of 43 kDa, and fused in sarcoma as very plausible prionoids contributing to the spread of the pathology. In this review, we will discuss the molecular and cellular mechanisms leading to the propagation of these prionoids in ALS.

## Teaser

Amyotrophic lateral sclerosis is a fatal neurodegenerative disease characterized by the loss of motoneurons in the brain and spinal cord. Disease onset is anatomically localized and progression occurs by spread to contiguous regions. Raoul *et al*. discuss the relevance of prion-like propagation as a pathogenic determinant in disease progression.

## Graphical Abstract

**Graphical Abstract:**
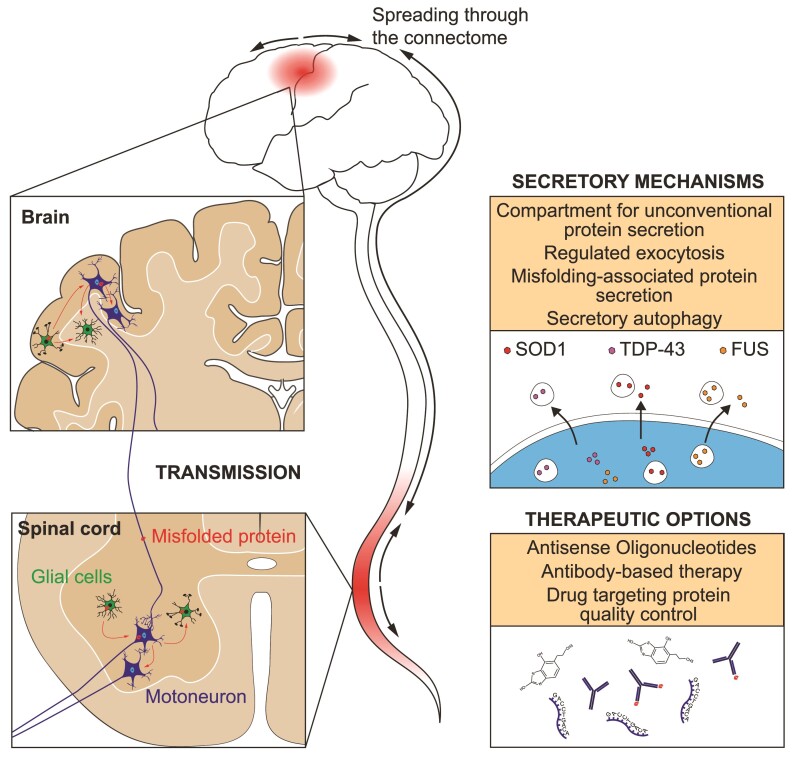

## Introduction

A common feature of neurodegenerative diseases is a scenario of events that leads to specific neuronal dysfunctions in a defined neuroanatomical region of the brain. The progressive failure of proteostasis in the CNS leads to the accumulation, aggregation and propagation of a subset of pathogenic proteins.^1,2^ These diseases commonly progress from focal sites and spread to neighbouring regions, as well as to the functionally connected, more distant areas of the CNS. The different stages of disease progression have been well described for Alzheimer’s disease, Parkinson’s disease and amyotrophic lateral sclerosis (ALS).^3,4^ In infectious prion diseases, the progression of neuronal degeneration in the CNS correlates with the spreading of a prion protein (PrP), and similar mechanisms of toxic protein propagation have been proposed for other neurodegenerative diseases, such as Alzheimer’s disease, Parkinson’s disease and ALS.^1,5^ The molecular basis of this proteotoxic spreading is the capacity of the deleterious scrapie PrP to pass its peculiar conformation onto other PrP species.^6^ Accumulating evidence indicates that α-synuclein, tau and Aβ peptides in Parkinson’s disease and Alzheimer’s disease have similar PrP properties and can seed and propagate within the CNS.^7,8^ For instance, prion-like proteins are found in insoluble deposits, referred to as amyloid fibrillary structures, such as α-synuclein in Lewy bodies in Parkinson’s disease and amyloid plaques in Alzheimer’s disease.^9,10^ The term prion-like proteins or prionoids is used because the associated neurodegenerative diseases are not likely to be infectious, as prion diseases are.^7–9^ In ALS, several proteins, including Cu/Zn superoxide dismutase 1 (SOD1), TAR DNA-binding protein of 43 kDa (TDP-43), and fused in sarcoma (FUS), harbour some features of prionoids.^10–12^ In this review, we will discuss recent findings on the spread of prion-like proteins in ALS and the current debates that highlight that there may also be a differential vulnerability of functionally interconnected neurons to neurotoxic prionoids, illustrating the complexity of pathogenic mechanisms.

### SOD1, TDP-43 and FUS proteinopathies in ALS

ALS is characterized by the selective loss of both upper and lower motoneurons that irremediably leads to progressive paralysis. This evolves rapidly into generalized wasting that irrevocably causes death by respiratory failure. The median survival time from the clinical onset is about 3 years. Approximately 90% of ALS cases are sporadic with largely unknown aetiology, but an increasing number of genes have been linked to the 10% of cases that show a familial inheritance of the disease.^13,14^ Some of these genes are also mutated in frontotemporal dementia (FTD), suggesting that these two diseases have overlapping physiopathological mechanisms.^15^ Both sporadic (sALS) and familial (fALS) forms of the disease are histologically characterized by the presence of multiple types of protein deposits in the CNS, which defines ALS as a proteinopathy. Currently, mutations in genes coding for C9ORF72, SOD1, TDP-43 and FUS are the most frequently found mutations in fALS.^13,14^ Whereas many genes (more than 30) are linked to fALS, only relatively few proteins (TDP-43, SOD1, FUS, C9ORF72 dipeptides, optineurin, ubiquitin and p62) are present in diverse types of inclusions in the spinal cords and brains of patients with ALS.^14,16^ Together, TDP-43 and FUS intracellular protein inclusions can be found in more than 90% of *post-mortem* tissues from patients with ALS and in more than 50% of tissues from patients with FTD, reflecting the pathological overlap that exists between these two disorders.^17^ SOD1 inclusions are predominantly found in patients with pure ALS with SOD1 mutations. Although mutant forms of TDP-43, FUS and SOD1 can be associated with distinct clinical manifestations,^18^ they share common pathogenic features that lead to ALS. The presence of these proteins in intracellular protein inclusions and aggregates in ALS might be explained by their low magnitude of solubility, which leads to an increased susceptibility to becoming insoluble when a slight cellular proteostasis disturbance occurs.^19,20^ Furthermore, TDP-43 proteinopathies are observed in other neurodegenerative diseases, such as Alzheimer’s disease and Parkinson’s disease.^21–23^ Structurally, although the inclusions are less well characterized in ALS than those found in Alzheimer’s disease or Parkinson’s disease, they are classified as non-amyloid or amyloid-like, as they do not fulfil the criteria of the highly ordered amyloid fibrils of proteins typically found in other neurodegenerative diseases, such as PrP, Aβ peptide, tau and α-synuclein.^9^

SOD1 is a cytosolic metalloenzyme of 154 amino acids (aas) involved in cell detoxification by catalyzing the conversion of the superoxide radical into oxygen and hydrogen peroxide.^24^*SOD1* was the first gene found to be linked to fALS, and mutations in this gene are found in ∼20% of cases.^25^ There are several lines of evidence indicating that the primary pathogenic mechanism of the disease is a toxic gain of function of SOD1 mutants. Although Sod1 null mice showed age-associated muscle denervation and increased susceptibility to oxidative stress, the loss of SOD1 function did not lead to typical ALS.^26,27^ In addition, ablation or overexpression of wildtype SOD1 does not affect SOD1 mutant-mediated motoneuron disease in mice.^28^ The concentration and enzymatic activity of SOD1 mutants observed in patients with fALS does not correlate with the age of onset or disease severity.^29,30^ The toxic gain of function seen with SOD1 mutants is associated with a higher propensity of these mutants to form aggregates when cellular quality control and protein catabolism decline.^28^ Several studies have shown an increase in misfolded wildtype SOD1, not necessarily in an aggregated state, in the spinal cord tissues of sALS and fALS patients.^31–35^ Misfolded SOD1 is present in upper and lower motoneurons and glia cells in sALS, supporting the fact that a global decline in protein homeostasis is a general feature in ALS. However, the contribution of misfolded wildtype SOD1 to pathology in sALS is still a matter of debate.^32,36^

TDP-43 is an RNA-binding protein (RBP), member of the heterogeneous nuclear ribonucleoprotein family, and is involved in several functions related to the regulation of RNAs, such as pre-mRNA splicing, mRNA stability and transport and translation.^23,37^ TDP-43 is a nuclear protein but may actively shuttle into the cytoplasm when associated with RNAs export complex.^37^ Several mutations in the *TARDBP* gene are linked with dominant fALS-FTD (4% of cases) and with <1% of fFTD cases.^38^ These TDP-43 mutants mislocalize in the cytoplasm, where they form aggregates.^39^ Both loss of function, due to TDP-43 nuclear depletion, and toxic gain of function, owing to proteostasis failure, contribute to the disease.^40,41^ In both sporadic ALS and FTD, wildtype TDP-43 is largely found in cytoplasmic inclusions that are mainly composed of post-translational modified TDP-43, including truncated, phosphorylated and ubiquitinated forms.^42–44^

FUS, like TDP-43, is an RBP involved in DNA repair, RNA splicing and RNA regulation, and is mainly localized in the nucleus.^35^ Mutations in *FUS* account for about 4–5% of fALS cases and occur very rarely in fFTD.^16,45^ Cytoplasmic aggregates of FUS are observed in the CNS of patients harbouring *FUS* mutations,^46,47^ but recently it has been shown that mislocalization and not necessarily aggregates of wildtype FUS might be present in sALS.^48^

### Basic cellular events of prion-like protein propagation

**Figure 1:**
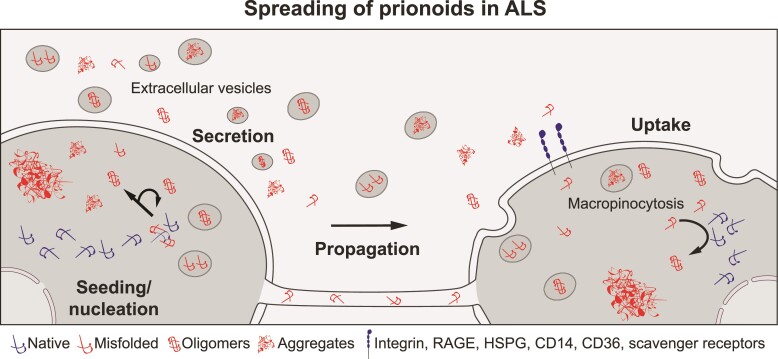
**Cellular events involved in the generation and spread of prionoids.** Seeding/nucleation: intracellular accumulation of cytoplasmic misfolded proteins induces prionoid seeding and nucleation in non-amyloid or amyloid-like structures termed ‘aggregates’. Oligomers are presumably intermediate structural protein species in the nucleation process. Propagation: mechanisms of cellular extrusion of misfolded proteins, comprising intercellular bridges like tubular nanotubes and unconventional secretion of proteins in a soluble state or contained in various secreted vesicles (lysosomes, endosomes, exosomes, extracellular vesicles, exophers).^49^ Cellular uptake: uptake of prionoids by macropinocytosis. This process seems to be regulated by several receptors that recognize extracellular protein aggregates.

The cellular pathways implicated in the propagation of prion-like proteins can be schematically divided into three steps: the release of prion-like proteins from cells, uptake by neighbouring cells and seeding and nucleation in recipient cells (Fig. 1). Although it is well documented that the spread of prion-like proteins in different neurodegenerative diseases involves interconnected neurons, it is, however, not always clear if the anatomical pattern of cellular alterations reflects the real spread of a toxic protein or if it is also due to a differential vulnerability of a neuronal population.^2^ The contribution of glial cells as an intermediary between populations of neurons is also to be considered in the dissemination of prion-like proteins.^50–52^ The mechanism of propagation of prion-like proteins in different neurodegenerative diseases may implicate different processes: proteotoxicity takes place extracellularly for Aβ and PrP and intracellularly for tau, α-synuclein and ALS-linked prion-like proteins (SOD1, TDP-43 and FUS).^53^ Eukaryotic cells have developed machineries based on chaperon-mediated quality control, proteasomal and autophagosomal protein degradation systems that prevent intracellular aberrant protein accumulation.^54,55^ However, the primary molecular events leading to deployment of misfolded harmful proteins in a subset of cells are still poorly understood, especially for sporadic neurodegenerative diseases. The progressive deterioration of proteostasis associated with ageing^56^ and poorly characterized environmental cellular stressors might favour intracellular seeding and nucleation of prion-like proteins, especially for those whose solubility threshold is already elevated in a defined cell.^9,19,55,57^ Abnormal and misfolded proteins, comprising different structural entities (monomers, oligomers and aggregates), can thus be removed by several mechanisms, such as via intercellular bridges and by active release or secretion via non-vesicular and vesicular transports (Fig. 1).^58–60^ Seminal works in *Caenorhabditis elegans* suggest that unconventional secretion induced by cellular stress of aberrant protein aggregates prevents cellular protein overload and furthermore promotes extracellular proteolysis of proteins that are usually not secreted.^61,62^ More specifically in neurodegenerative diseases, prionoids such as misprocessed Aβ forms and mutant PrP escape endoplasmic reticulum (ER) quality control and are exported via the vesicular conventional secretory pathway, whereas α-synuclein, tau, TDP-43, FUS and SOD1 are cytoplasmic or nuclear leaderless proteins, and their extracellular release involves what is called unconventional secretory pathways.^63–65^ Special attention has been paid in recent years to the rules of different types of secreted vesicles (lysosomes, endosomes, exosomes, extracellular vesicles and exophers)^66^ in prionoid propagation, and there is some molecular evidence that cytoplasmic misfolded proteins are mistargeted to these different vesicles.^67,68^ Subsequently, absorption of prionoids by neighbouring or contacting cells allows their propagation in the CNS, an event that is called cross-seeding (Fig. 1). The cellular pathways involved in the internalization of ALS prion-like proteins in a recipient cell are still poorly characterized, but some specific receptors have been identified (Fig. 1). After cellular entry, prion-like proteins reach the cytosol by a still unknown mechanism and initiate a new cycle of seeding/nucleation (Fig. 1). The molecular mechanisms of the cross-seeding and nucleation of SOD1, TDP-43 and FUS are not yet fully characterized, as they do not behave like classical amyloids. Finally, infected cells will *de novo* transfer prion-like proteins to other ‘naïve’ cells. The key steps of prion-like protein propagation will be described in more detail for SOD1, TDP-43 and FUS.

### Cellular and molecular mechanisms potentially involved in ALS prionoid propagation

#### Secretion pathways of SOD1, TDP-43 and FUS

**Figure 2:**
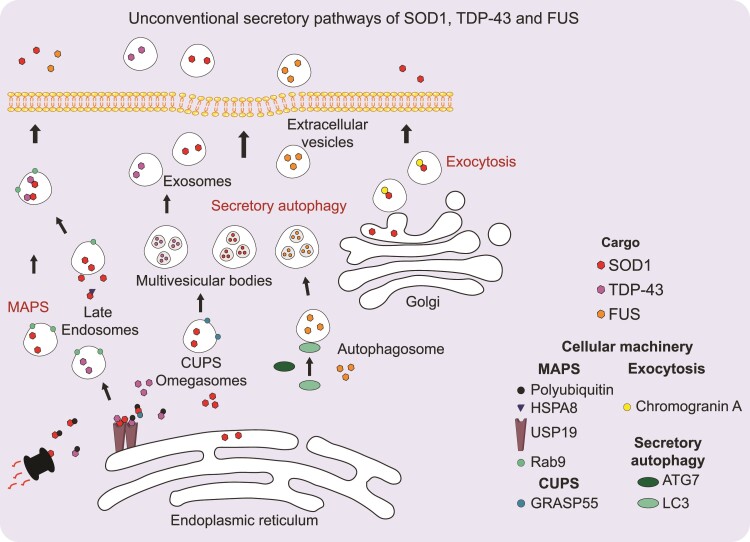
**Unconventional secretion pathways of SOD1, TDP-43 and FUS.** The cytoplasmic cargos SOD1, FUS and TDP-43, are represented by red, orange and purple circles, respectively. SOD1 is released either by a specific secretory pathway for cytoplasmic ubiquitinated misfolded proteins called MAPS^65^ or by an autophagic-like secretory pathway involving a specialized organelle called CUPS.^71^ SOD1 mutants are also secreted via exocytosis from motoneurons.^72^ The MAPS pathway seems also to promote wildtype TDP-43 secretion. FUS has, for instance, only been detected in extracellular vesicles from LC3^+^ autophagosomes.^73^ MAPS involves the targeting of cargos in Rab9^+^ (green circles) late endosomes allowing protein secretion in a vesicles-free form.^65^ This process depends on the binding of HSPA8 (marine blue triangle) and the deubiquitinase USP19 (brown stick) to polyubiquitinated (black circle) proteins. The CUPS-dependent pathway involves the Golgi reassembly-stacking protein (GRASP55) that appears to shuttle between the Golgi and CUPS.^71^

A large proportion of the prion-like proteins associated with various neurodegenerative diseases are leaderless proteins: α-synuclein, tau and huntingtin, as well as SOD1, TDP-43 and FUS. The secretion of proteins that do not bear a signal peptide has received particular attention for decades but remain poorly understood.^63,69^ Complex and diverse pathways of unconventional secretion, which are not linked to the canonical ER–Golgi secretory pathways, have emerged.^63,70^ The type of unconventional secretion varies depending on the target protein, and different pathways have been described for ALS-linked leaderless proteins (Fig. 2).

Before the discovery of fALS-linked mutations in the *SOD1* gene, studies in mouse bone marrow cell cultures had revealed regulated secretion of wildtype SOD1.^74,75^ Several studies have demonstrated the secretion of wildtype SOD1 by various cell types.^74,76–78^ SOD1 export is increased by membrane depolarization in excitable neuroendocrine cells,^74^ occurs through an ATP-dependent mechanism in neuroblastoma cells,^79^ and could be related to secretory autophagy in yeast.^77^ Indeed, during nutrient starvation of yeast, SOD1 is targeted to a specific cellular location called the compartment for unconventional protein secretion (CUPS), which is similar to the pre-autophagic compartment omegasome in mammalian cells.^77,80^ This CUPS-associated export of SOD1 is also dependent on some components of the endosomal sorting complexes required for transport. A diacidic motif, D76-E77, in SOD1 is required for this unconventional secretion, and interestingly, mutations (D77V, D77Y) in this motif are found in fALS.^77,81,82^ However, the SOD1^G93A^ mutation also leads to a decreased secretion compared with wildtype SOD1 that is greater than when the mutation impacts the diacidic motif itself.^77^

Other studies have also described unconventional secretion of wildtype and mutant SOD1 via exosomes (Fig. 2).^83–85^ The contribution of wildtype SOD1 secretion to cell survival and response to oxidative stress still needs to be clarified.^86,87^ Interestingly, SOD1^G93A^ mutant expression in astrocytes induces a global decrease in secreted proteins but an increase in protein content in the exosomal fraction compared with wildtype SOD1.^84^ This observation could be a result of an increased unfolded protein response.^86,88^ The abundance of SOD1^G93A^ in comparison to wildtype SOD1 appears to paradoxically decrease in exosomes but increase in the non-exosomal fraction.^84^ The exosomes from SOD1^G93A^ mouse astrocytes are toxic to motoneurons, but it is not known whether this toxicity is directly due to exosomal SOD1^G93A^.^84^ Secretion of SOD1 mutants can also occur through a more ‘conventional’ secretory pathway.^72,89–91^ Identified as a specific interaction partner of SOD1 mutants, the neuroendrocrine secretory protein chromogranin A recognizes only mutant SOD1 in motoneurons and targets it to exocytosis vesicles.^72^ How chromogranin transfers mutant SOD1 in exocytotic vesicles is still unknown but seems to be dependent on interactions with the Golgi apparatus. Furthermore, SOD1 import into the ER has been shown to be a post-translational and signal recognition particle-independent mechanism.^89^ Although post-translational ER targeting of proteins appears to be more frequent than expected, there are few examples of leaderless small proteins being targeted in this way, with the exception of proteins smaller than 50 aas.^92,93^ Another unconventional secretion pathway specifically for misfolded proteins, called misfolding-associated protein secretion (MAPS), is involved in wildtype SOD1 release.^94^ MAPS involves the ER-resident ubiquitin-specific protease 19 (USP19) deubiquitinase that redirects proteins destined for the proteasomal pathway to secretory endosomes (Fig. 2).^94,95^ Many cytosolic misfolded proteins, including mutant SOD1, might follow this unconventional secretion pathway. The USP19-dependent pathway involves the HSPA8 heat shock protein that targets misfolded proteins to USP19 (Fig. 2).^94,95^

The functional implications of these different unconventional secretions have been poorly exploited in SOD1-linked ALS mouse models, but some pioneering works have explored the secretion and intercellular transfer of SOD1 *in vivo*.^96^ In chimeric transgenic mice, transfer of both human mutant SOD1 and wildtype SOD1 between motoneurons and oligodendrocytes has been observed.^96^ It appears that transferred mutant SOD1 is not aggregated and does not co-aggregate with differently tagged human SOD1 in acceptor cells. This cell-to-cell protein transfer does not seem to be specific for mutant SOD1, as transfer of overexpressed wildtype human SOD1 and GFP between motoneurons and oligodendrocytes also occurs.^96^ Cellular mechanisms of SOD1 transfer *in vivo* are still unknown, but interestingly, motoneuron-specific deletion of *autophagy related 7* (*Atg7*) in *SOD1^G93A^* mice has peculiar effects on ALS pathogenesis.^97^ ATG7 is well known to be involved in secretory autophagy, an unconventional secretory pathway that facilitates the export of leaderless proteins.^98^ Aggregation of the autophagy receptor p62 into skein-like inclusions is first observed in spinal motoneurons and then in interneurons in SOD1-mutant mice. Inhibition of autophagy in motoneurons delays the formation of p62 inclusions and accumulation of misfolded SOD1 in interneurons. Aggregation of p62 in interneurons occurs concomitantly with astrogliosis and microgliosis. These results highlight the non-cell-autonomous effect of motoneuron autophagy on interneurons and glial cells, maintaining the possibility that direct spreading of misfolded SOD1 from motoneurons to interneurons occurs.^97^ It will be of interest to explore whether this phenotype is linked to a decrease in human SOD1 propagation, as has been observed for Aβ peptides in Alzheimer mouse models crossed with ATG7 knockout mice.^99^

The transfer of TDP-43 oligomers from cell to cell has been shown in primary neurons using protein complementation assays.^100,101^ This unconventional secretion of TDP-43 is induced by oxidative stress and mediated by exosomes (Fig. 2).^102,103^ Moreover, secretion of wildtype and mutant TDP-43 in exosomes is also observed in basal conditions in transformed cells and might be promoted by secretory autophagy (Fig. 2).^102^ Mutated TDP-43 has also been found in exosomal fractions purified from the brains of patients with ALS and the spinal cords of *TDP-43^A^315^T^* transgenic mice.^102^ It is noteworthy that the inhibition of exosome biogenesis by GW4869, an inhibitor of sphingomyelinase, or the inhibition of exosome release by Rab27 silencing in neuro2a cells increases cytoplasmic and nuclear aggregation of phosphorylated TDP-43. This suggests that release and biogenesis can be coupled events.^100,101^ However, inhibition of exosome release with GW4869 has minor effects on the phenotypic behaviour of *TDP-43^A315T^* transgenic mice.^102^ Wildtype TDP-43 can also be unconventionally secreted by MAPS in an exosome-independent manner *in vitro* (Fig. 2).^94^ The interconnection between the MAPS pathway and secretory autophagy remains unknown.

Although FUS (as well as SOD1 and TDP-43) has been found in plasma microvesicles and exosomes from patients with ALS, the mechanism of unconventional FUS secretion remains poorly documented.^104^ Recently, proteome characterization of extracellular vesicles, which depend on microtubule-associated protein 1A/1B-light chain 3 (LC3) and ATG8 proteins for their secretion, has notably revealed a strong enrichment in RBP proteins, including FUS (Fig. 2).^73^ In basal conditions, RBPs associated with mRNAs account for a large proportion of secreted proteins.^73^ Whether this pathway implicating components of the autophagy machinery is increased in response to cellular stress remains an open question, but it provides a molecular basis for the unconventional secretion of FUS and other ALS-related RBPs.^105^^(p3)^

#### Cellular uptake of extracellular prionoids

Uptake of extracellular SOD1 aggregates has been extensively studied in cultures of neuronal and non-neuronal cells expressing different ALS-related mutant SOD1.^106^ Studies revealed that extracellular mutant SOD1 aggregates can enter cells and nucleate endogenous mutant but not wildtype SOD1.^106,107^ It was subsequently suggested that SOD1 aggregates enter acceptor cells by macropinocytosis in order to reach the cytosol.^106^ Macropinocytosis is a form of non-selective endocytosis generally induced by growth factors and used by cells to internalize large numbers of soluble macromolecules or particles too large for other forms of endocytosis.^108,109^ Because macropinosomes are characterized by the absence of a coat structure to guide their formation and by their heterogeneous size and shape, they give non-phagocytic cells the ability to take up large particles in response to an external stimulus.^109^ Subsequent work has revealed that SOD1 aggregates stimulate this mechanism to enter the cells.^110^ However, how SOD1 aggregates stimulate macropinocytosis remains unclear.^110^ It has been shown that the Rho-ROCK1-LIMK1 signalling pathway regulates extracellular SOD1 macropinocytosis via cofilin activation.^111^ In addition, by displaying heparan sulfate proteoglycans, receptor for advanced glycation end-products, CD36, integrins and receptor tyrosine kinases, neurons can trigger macropinocytosis following the binding of extracellular protein aggregates (Fig. 1).^112^ In microglial cells, the binding and internalization of extracellular SOD1 aggregates involve lipid raft formation, scavenger receptors and CD14.^113,114^

#### Seeding and nucleation of SOD1, TDP-43 and FUS

##### Self-perpetuation of SOD1 aggregation

Once ALS prionoids enter the cell and reach the cytoplasmic compartment, they must escape cellular quality control and degradation to co-aggregate with their wildtype or mutant counterpart (Fig. 1). The seeding and nucleation mechanisms of ALS prion-like proteins are still not known, but there are striking fundamental structural differences between SOD1 and the two RBPs, TDP-43 and FUS. Whereas folded wildtype SOD1 is stable, TDP-43 and FUS contain intrinsic disorder domains (IDRs) that promote their self-assembly and lead to a phase transition.^115^ Thus, in contrast with SOD1, TDP-43 and FUS oscillate easily between different oligomeric structural states that can make them more prone to aggregation.^115,116^

The first studies to suggest a prion-like activity of SOD1 *in vivo* showed that the expression of human wildtype SOD1 aggravates the phenotype of mice expressing ALS-linked mutant SOD1.^117–119^ In addition, the co-expression of a truncated form of mutant SOD1 with wildtype SOD1 in mice triggered the induction of wildtype SOD1 aggregation by mutant SOD1.^120^ Soluble heterodimers or oligomers of wildtype and mutant SOD1 can also be detected in cell culture.^121,122^*In vitro*, several studies have shown the seeding and nucleation of wildtype SOD1 in the presence of mutant SOD1.^123–129^ The nucleation conditions of SOD1 *in vitro* necessitate reducing and denaturing agents to favour metal-deficient apoSOD1 and formation of amyloid-like fibrils. This suggests that newly synthetized and unfolded SOD1 is the species that is most likely to form amyloid-like fibrils in the presence of misfolded SOD1. The folding of SOD1 as a fully mature homodimeric metalloenzyme has been well studied *in vitro* and appears to be a slow process.^130–134^ Indeed, SOD1 in an unfolded state has a longer lifetime than most other proteins in a test tube, therefore increasing the probability of it forming aberrant oligomeric structures during folding.^131,132,135^ SOD1 bears several peptide sequences at both N- and C-terminus that have been implicated in fibril formation.^136^ Furthermore, mutations in these peptide segments found in fALS often enhance the aggregation propensity of SOD1.^136^ One particular sequence (aas 28–38) adopts a peculiar corkscrew-like structure that is indispensable for the formation of soluble toxic oligomers and likely leads to seeding of unfolded SOD1.^137,138^ In addition, nucleation of wildtype SOD1 can induce the formation of intermolecular disulfide bonds in the highly reducing environment of the cytoplasm.^106,129,135,139^ However, intermolecular disulfide bonds are not necessary for the initiation of seeding but could be involved in the sequestration of potentially toxic oligomeric SOD1.^140^ Other evidence suggests that the formation of these hyperoxidized SOD1 aggregates takes place in the ER, which provides a more suitable environment for disulfide bond formation.^91^

The cell-to-cell transmission of misfolded mutant SOD1 was evidenced in studies in which preformed SOD1 aggregates were added to cell cultures.^106,129^ While the extracellular aggregates applied were readily proteolyzable, the intracellularly induced aggregates appeared to be persistent and transferred from cell to cell.^106^ This transmission is more likely due to endogenous cycles of secretion, internalization and nucleation of wildtype SOD1 than to the persistence during successive cell divisions of the SOD1 aggregates that were initially added to the cultures. This suggests that self-amplification either during cell division or/and passage through the extracellular medium is a prerequisite for the cellular spreading of SOD1.^106,107,129^ Furthermore, in cell culture, expression of other pathogenic proteins such as FUS and TDP-43 induces wildtype SOD1 misfolding that can be transmitted to naïve cells in a prion-like fashion.^85,141^

##### Self-perpetuation of mutant TDP-43 and FUS aggregation

**Figure 3:**
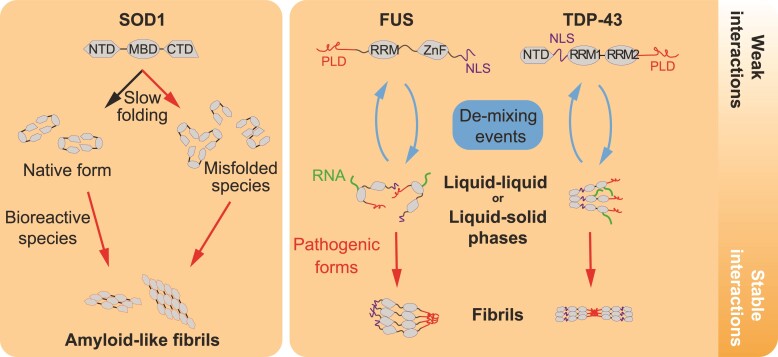
**Pathological aggregations of SOD1, FUS and TDP-43.** Schematic view of pathways leading to the aggregation of SOD1 and the two RBPs, FUS and TDP-43. SOD1 aggregation results in the accumulation of misfolded oligomeric or monomeric species during the folding process that can be due to intrinsically disordered structure (fALS mutations) and defects of the protein quality control and folding machineries. Alternatively, the properly folded SOD1 dimer might be also destabilized due to deleterious physicochemical perturbations (e.g. reactive oxygen species). FUS and TDP-43 aggregation might be the result of an imbalance in their oscillations between disordered monomers and highly ordered oligomeric structures. These structural properties give rise to labile phase transitions from liquid to liquid (liquid droplets) or to liquid to solid (hydrogel). ALS-causing mutations, cellular dysregulation (RNA metabolism, trafficking defects…) favour aberrant and irreversible phase transitions (liquid/solid, solid/solid) producing fibrils (seeding). NTD (N-terminal domain), MBD (metal binding domain), RRM (RNA recognition motif), ZnF (zing finger domain) and NLS (nuclear-localization sequence).

At its N-terminus, TDP-43 is composed of a dimerization domain (N-terminal domain), a nuclear-localized sequence and two RNA recognition motifs (RRMs).^110,141,142^ The C-terminal part has two IDRs and a glycine-rich domain involved in multivalent weak intermolecular interactions.^115^ Like other proteins containing IDRs, also called prion-like domains, TDP-43 is well known to self-assemble and creates a separate phase in membrane-less compartments like stress granules (Fig. 3).^143,144^ The two domains RRM1 and RRM2 of TDP-43 recognize GU-rich RNA sequences on several pre-mRNAs.^142,144^ mRNA concentrations seem to be key regulators of TDP-43 amyloid-like structures. Indeed, TDP-43 aggregates are formed when cytoplasmic mRNA levels decrease.^145,146^ This property explains why TDP-43 and FUS form aggregates predominantly in the cytoplasm rather than in the nucleus.^145^ ALS-causing mutations in TDP-43 have been shown to increase the susceptibility of TDP-43 to forming cytoplasmic insoluble oligomers (Fig. 3).^147,148^ Widely recognized to occur in ALS and FTD, wildtype TDP-43 labile condensates can also be transformed into insoluble aggregates.^146^ The circumstances that promote wildtype TDP-43 cytoplasmic aggregates, as well as their pathological relevance, are a matter of intense debate.^149,150^ In non-pathological conditions, cytoplasmic inclusions of TDP-43 amyloid-like structures called myo-granules have been found to be essential for myotube formation in skeletal muscle.^151,152^ Interestingly, myo-granules are mostly insoluble, contain sarcomere mRNAs and can seed monomeric TDP-43 *in vitro*.^152^ This means than even cytoplasmic TDP-43 amyloid-like oligomers can be formed and disassembled in some cell types and that failure of disaggregation might be the clue to cellular intoxication. Furthermore, an elegant study using real-time microscopy revealed that TDP-43 undergoes highly dynamic mixing and de-mixing into droplets in the cell nucleus (Fig. 3).^40^ De-mixing is a new term for the segregation and oligomerization of TDP-43 that well defines this physicochemical reaction that is based on weak protein–protein interactions.^40^ The functional consequence of this condensation process driven by the coordination of the two IDRs is to concentrate a subset of pre-RNAs in the nucleus.^143,144^ However, when TDP-43 de-mixing occurs in the cytoplasm, TDP-43 is depleted in the nucleus, leading to cell death.^40^ Mislocalization and aggregation of TDP-43 can be induced independently of stress granule formation by nuclear import disruption and by a broad range of cellular stresses, including proteasomal inhibition, ER misfolding and reactive oxygen species.^40,153–155^ Importantly, extracellular TDP-43 fibrils can induce irreversible cytoplasmic TDP-43 de-mixing reminiscent of prion-like activity as it has been suggested earlier.^40,100,156^ As exogenous FUS fibrils but not SOD1 fibrils also induce similar TDP-43 de-mixing, it is not clear if TDP-43 and FUS fibrils directly nucleate endogenous TDP-43.^40^ Interestingly, cytoplasmic TDP-43 myo-granules, similar to structures observed in injured neurons, are formed in differentiated myotubes during muscle regeneration.^152^ However, TDP-43 in myo-granules does not seem to be post-translationally modified as observed in cytoplasmic TDP-43 aggregates found in neuronal tissues from sALS.^152^ It is unknown why cytoplasmic TDP-43 de-mixing in myotubes is reversible and safe, whereas it induces cell death when induced by RBP fibrils in neurons.^152^ Though the properties of TDP-43 aggregation in cells are becoming better understood, the structural and biophysical determinants involved in the seeding and nucleation of TDP-43 remain largely uncharacterized (Fig. 3).

Similar to TDP-43, FUS has an IDR located at the N-terminus that promotes liquid–solid phase transition.^157–159^ The prion-like IDR domain can self-oligomerize and may promote amyloid-like structures of FUS in the nucleus as well as in the cytoplasm.^115,159–161^ These structural properties allow FUS to undergo liquid–solid phase transition in the event of increased FUS concentrations.^158,161,162^ fALS FUS mutations, mainly localized in the IDR, have the tendency to increase the speed of the liquid–solid transition.^159,160,163^ However, it is not known if the amyloid-like oligomer formation of FUS might be linked to the spread of disease progression in fALS or sALS.

### Physiopathological evidence of SOD1 and TDP-43 prion-like propagation in mice

**Figure 4:**
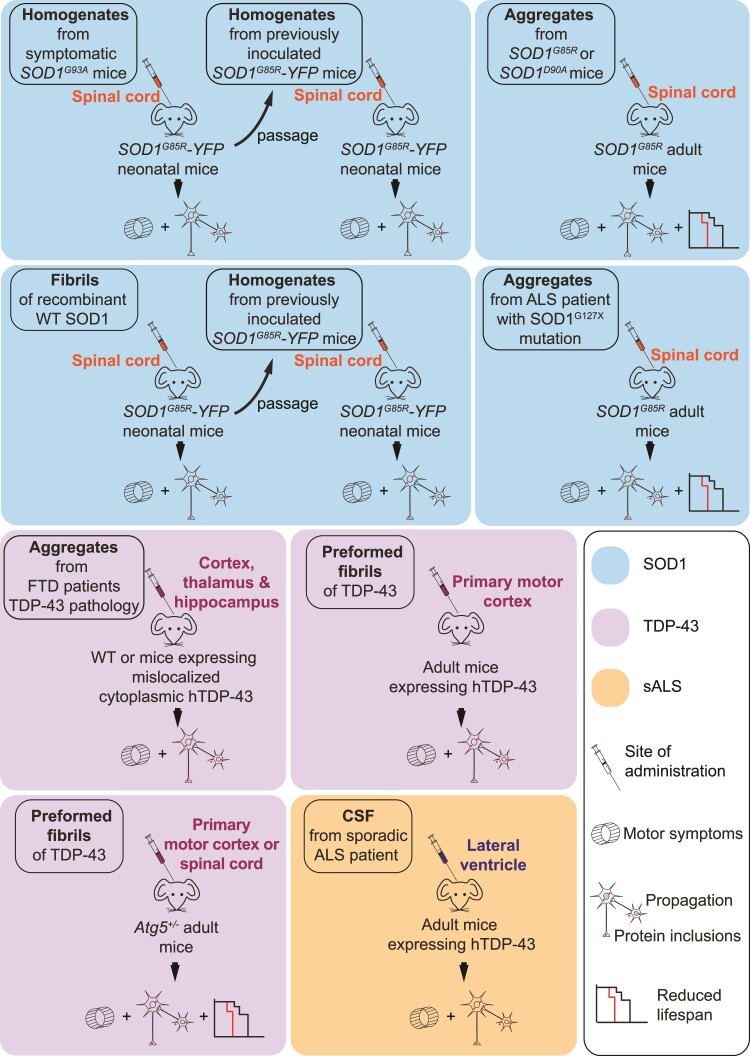
**Experimental paradigms demonstrating the prion-like properties of SOD1 and TDP-43 in mice.** This schema is a compilation of several published results obtained by the administration of misfolded SOD1 and TDP-43 aggregates in different mouse models. The nature of protein extracts (crude homogenates, purified proteins, recombinant proteins), the route of delivery and the genetic background of injected mice are indicated. For the observed physiopathological outcome, three criteria are mentioned: the levels of protein inclusions/aggregates in the CNS, the motor symptoms evaluated by behaviour tests and the lifespan of injected mice.

Initial evidence of prion-like propagation of human mutant SOD1 was revealed in *in vivo* experiments in which protein homogenates obtained from symptomatic *SOD1^G93A^* mice were injected into the spinal cords of non-symptomatic *SOD1^G85R^-YFP* mice (Fig. 4).^164^ Injected mice displayed motor symptoms and reduced lifespan, but the observed penetrance was about 60%. In a second passage, injection of homogenates of the spinal cords of SOD1^G85R^-YFP mice that had been inoculated previously and become paralyzed produced the same symptoms in mice but with full penetrance.^164^ The same process of passages has been used with different strains of SOD1, resulting in distinct pathologies.^31^ Another study showed that transgenic mice expressing different SOD1 mutants could produce distinct types of SOD1 aggregate strains.^165^ Two of these SOD1 strains (G85R and D90A) were then injected into the lumbar spinal cords of asymptomatic animals, resulting in SOD1 aggregation and rostral spreading of the pathology (Fig. 4).^166^ A rapid induction of motor deficits and premature motoneuron death were then observed.^166^ This work suggests the existence of multiple mutant SOD1 strains that are able to seed and spread in mutant SOD1 transgenic mice. In another study, protein homogenates from *SOD1^G^*^127*X*^ patients were injected into the spinal cords of SOD1^G85R^-expressing mice.^167^ The animals then developed fatal ALS-like pathology and exhibited spreading of SOD1 aggregation.^167^ Additionally, injection of mutant SOD1 seeds into the sciatic nerve induced disease spread in the peripheral nervous system as well as in the spinal cord.^167^ However, other types of peripheral administrations (intraperitoneal and intramuscular) of highly potent SOD1 seeds do not seem to allow the spread of ALS pathology in the CNS.^168^ In contrast with other prion-like pathogenic proteins found in Alzheimer’s disease or Parkinson’s disease, no evidence of an ALS pathology supported by wildtype human SOD1 spread have been revealed.^169^ Indeed, recent work reveals that transgenic mice overexpressing wildtype SOD1 appear to be resistant to misfolded mutant SOD1 seeds.^169^ Together, these results show that mutant SOD1 aggregates can transmit and propagate ALS-like dysfunctions when they reach the CNS.

The potential transmissibility and spreading of TDP-43 proteinopathy has been revealed by a study in which insoluble protein extracts from the brains of patients with sporadic FTD were injected into the CNS of mice (Fig. 4).^170^ Pathological TDP-43 spreading was progressively observed in a spatio-temporal manner in different brain areas, supporting cell-to-cell transmission throughout the CNS.^170^ The CSF as a route for spreading of pathology has been explored by a study in which the CSF of patients with sALS was intracerebroventricularly injected into mice expressing wildtype human TDP-43.^171^ Motor, cognitive and pathological changes were observed in the transgenic mice that received ALS CSF. These ALS-like alterations also suggest a threshold effect for seeding of TDP-43. Indeed, the motor phenotype in response to ALS CSF infusion was more pronounced in mice expressing human TDP-43 than in wildtype mice, and cytoplasmic aggregation of TDP-43 was observed only in transgenic mice and not in wildtype mice. Until recently, it was still unknown whether different strains of TDP-43 might support multiple TDP-43 proteinopathies in the CNS, but recent studies have provided new evidence about the spreading of TDP-43 preformed fibrils in human TDP-43 and in *Atg5^+/−^* mice. These studies observed spreading all along the corticospinal tract in a time-dependent manner, which supports Braak’s model.^172–174^ In the case of FUS, although it can behave like a prionoid *in vitro*, no *in vivo* experimental evidence currently exists that demonstrates the spread of FUS proteinopathy.

### Limits and caveats of prion theory in ALS

Despite the evidence presented above, some points continue to cast doubt on the importance of prion-like mechanisms for transmission and spreading in ALS. A fundamental difference between ALS prionoids and PrP or prionoids including Aβ, α-synuclein and tau is that the latter can form amyloid fibrils in human CNS. Amyloid fibrils are extracellular deposits of a fibrillary protein with a β-sheet secondary structure.^175^ Biochemical properties of amyloids such as seeding and protease resistance obviously facilitate their dissemination. In addition, the natural secretion of PrP and Aβ peptides that occurs facilitates their dissemination even further. For now, no kind of disease transmission between individuals has been documented in the case of ALS, whereas an observation of this kind has been reported for Alzheimer’s disease.^176^ Furthermore, inoculation of brain homogenates from patients with ALS into the brain of non-human primates did not reveal any disease transmission.^177^ In this large-scale study, similar conclusions were made for other non-spongiform neurological diseases, including Alzheimer’s disease and Parkinson’s disease. The phylogenetic distance between human and non-human primates might explain the failure to transmit non-spongiform diseases. Recently, inoculation of brain homogenates from patients with Alzheimer’s disease into mouse lemurs led to the spreading of Aβ and tau pathology in different brain regions associated with cognitive defects.^178^ It should also be noted that a more detailed histopathological study and follow-up of the motor capacity of the primates could have helped to reveal potential transmission. Another study reported that intramuscular or intraperitoneal inoculation of spinal cord homogenates from endstage *SOD1^G85R^* mice that contained SOD1 aggregates did not transmit SOD1 aggregation to the CNS or peripheral tissues of recipient mice.^168^ It is noteworthy that the SOD1 aggregates were found to be highly sensitive to protease degradation, which is not the case for highly transmissible species, such as the scrapie isoform of PrP. This observation could explain the reduced ability to transmit the disease-associated aggregation from the peripheral tissues to the CNS. Finally, a recent study showed that genetic ablation of the *SOD1^G37R^* transgene in corticofugal projection neurons, which prevent corticospinal neuron degeneration, does not influence disease onset, progression, life expectancy or spinal motoneuron survival. Interestingly, the corticofugal projection neuron-selective excision of mutant SOD1 did not influence the levels of misfolded mutant SOD1 in the spinal cord.^179^ These results led the authors to conclude that corticofugal disease propagation is not mediated by a prion-like mechanism but rather by cortical hyperexcitability.^179^ Their findings do not seem to be consistent with Braak’s staging scheme,^174^ where mutant SOD1 in corticofugal neurons may not be essential for disease propagation, although a prion-like mechanism may still operate in the spinal cord.

### Clinical and therapeutic considerations

The eventual progression of ALS from an initial site to distant regions in the CNS is well described. The disease starts in one part of the body, in the vast majority of cases in one hand, one foot or as a speech disturbance. Within months or years, the disease spreads to other parts of the body and gradually becomes more debilitating. It has been noted that contiguous areas are more rapidly involved in the disease process of ALS than non-contiguous areas.^180^ Importantly, pathological studies in humans have confirmed this progressive and stepwise spreading of ALS, following a model of corticofugal spread.^174^ Although neuronal involvement progresses at different rates from one patient to another, it nevertheless follows a similar sequence between patients. In animal models, and particularly in mutant SOD1 mice, the same phenomenon has been described and follows anatomical pathways.^181^ To date, in clinical neurology, few other potential explanations have been proposed to interpret the progression of ALS. One interesting suggestion was that a progressive and stepwise activation of microglia might also occur, secondary to initial prionoid spreading.^182^ A study evaluated this microglial hypothesis by use of selective Cre recombinase-mediated SOD1 deletion in microglial cells via targeting of the CD11b promoter. The impact on disease progression was highly significant; the lifespan of the mice improved by 75 days.^182^ In another study, the addition of SOD1 aggregates to microglial cells in culture triggered their activation.^114^ This study suggests that mutant SOD1 aggregates can contribute to disease progression by promoting microglial activation and subsequent release of toxic factors to motoneurons.^114^ Targeting both extracellular and intracellular misfolded SOD1, anti-misfolded SOD1 antibodies have been successfully used as a potential immunotherapeutic approach in mice.^183,184^ More recently, peripheral administration of an antibody specifically recognizing misfolded SOD1 delayed disease onset, improved the decline of motor function, and increased the lifespan of *SOD1^G37R^* and *SOD1^G93A^* mice.^185^ An alternative approach has been the development of an ALS vaccine targeting a conformation-specific epitope. This aimed to reduce the burden of extracellular SOD1 and has provided therapeutic benefits in ALS mice.^186,187^ From a therapeutic point of view, the discovery of prionoid spreading in people with ALS opens perspectives that cover a large number of pathways. Proteostasis is a particularly important mechanism to consider. It has been shown that riluzole, the only marketed drug for ALS that has a positive effect on ALS patients’ survival, enhances the expression of cytosolic chaperons HSP70 and HSP90 by increasing the heat shock protein response.^188^ Clinical trials are underway to evaluate a series of drugs acting on proteostasis, such as arimoclomol,^189^ fasudil^190^ and memantine.^191^

Other promising approaches that tackle prionoid activity at the root consist of silencing SOD1. The proof-of-principle of SOD1 gene silencing in mice with virally delivered shRNA has already been established^192,193^; more recently it was shown that subpial delivery of adeno-associated adenovirus serotype 9 prevented disease onset when injected before ALS onset, and blocked disease progression when delivered after onset.^194^ One promising therapeutic approach is gene modulation by antisense oligonucleotides (ASOs). ASOs have been given intrathecally to patients with ALS; treatment has been shown to be well tolerated and result in significantly lowered SOD1 levels in the CSF.^195^ A phase I/II trial with torfersen ASO in 50 patients with SOD1 mutations not only revealed a good tolerance profile, but also suggested that the clinical impact of this therapy is worth exploring in a phase III trial.^196^ In October 2021, results of the tofersen phase III study (VALOR) were presented at the American Neurological Association annual meeting (https://investors.biogen.com/). Although the primary efficacy endpoint as determined by the revised ALS functional rating scale was not met, secondary measures, including SOD1 CSF and plasma neurofilament light chain (a neuronal degeneration marker) levels, reached the goal of change from baseline. Trends towards a benefit for respiratory function, muscle strength and quality of life are further promising signs. One of the possible directions that could lead to a better outcome with this treatment is earlier and longer intervention, and Biogen is currently running a phase III trial (ATLAS) in presymptomatic SOD1 mutation carriers (https://clinicaltrials.gov/ct2/show/NCT04856982).

## Conclusion

Perhaps more so than in other neurodegenerative diseases, the field of ALS research is incredibly dynamic, as functionally and structurally unrelated new genes involved in fALS are being discovered each year and illuminating novel molecular and cellular fundamental mechanisms. A challenge for the scientific community remains as to how all these diverse pathological genetic dysfunctions lead to a disease that is quite homogenous. In this context, the study of the spread of prionoids in ALS remains in its infancy and might offer new therapeutic approaches. Preventing disease progression in ALS is an important therapeutic strategy that will be boosted by our increased knowledge about the molecular and cellular mechanisms that lead to the dissemination of neurotoxic prionoids.

## Data availability

Data sharing is not applicable to this article as no new data were created or analysed in this study.

## Abbreviations

aas =: amino acids
ATG7 =: autophagy related 7
CUPS =: compartment for unconventional protein secretion
ER =: endoplasmic reticulum
fALS =: familial ALS
FTD =: frontotemporal dementia
FUS =: fused in sarcoma
IDR =: intrinsic disorder domain
LC3 =: microtubule-associated protein 1A/1B-light chain 3
MAPS =: misfolding-associated protein secretion
PrP =: prion protein
RBP =: RNA-binding protein
RRM =: RNA recognition motif
sALS =: sporadic ALS
SOD1 =: Cu/Zn superoxide dismutase 1
TDP-43 =: TAR DNA-binding protein of 43 kDa
USP19 =: ubiquitin-specific protease 19

## References

[1] Jucker M , Walker LC. Propagation and spread of pathogenic protein assemblies in neurodegenerative diseases. Nat Neurosci. 2018;21(10):1341–1349. 30258241 10.1038/s41593-018-0238-6 PMC6375686

[2] Vaquer-Alicea J , Diamond MI. Propagation of protein aggregation in neurodegenerative diseases. Annu Rev Biochem. 2019;88(1):785–810. 30917002 10.1146/annurev-biochem-061516-045049

[3] Braak H , Braak E. Neuropathological stageing of Alzheimer-related changes. Acta Neuropathol. 1991;82(4):239–259. 1759558 10.1007/BF00308809

[4] Goedert M . Alzheimer’s and Parkinson’s diseases: The prion concept in relation to assembled A, tau, and -synuclein. Science. 2015;349(6248):1255555. 26250687 10.1126/science.1255555

[5] Prusiner SB . Biology and genetics of prions causing neurodegeneration. Annu Rev Genet. 2013;47:601–623. 24274755 10.1146/annurev-genet-110711-155524 PMC4010318

[6] Carlson GA , Prusiner SB. How an infection of sheep revealed prion mechanisms in Alzheimer’s disease and other neurodegenerative disorders. Int J Mol Sci. 2021;22(9):4861. 34064393 10.3390/ijms22094861 PMC8125442

[7] Kara E , Marks JD, Aguzzi A. Toxic protein spread in neurodegeneration: Reality versus fantasy. Trends Mol Med. 2018;24(12):1007–1020. 30442495 10.1016/j.molmed.2018.09.004

[8] Wong YC , Krainc D. α-Synuclein toxicity in neurodegeneration: Mechanism and therapeutic strategies. Nat Med. 2017;23(2):1–13. 10.1038/nm.4269 PMC8480197 28170377

[9] Chiti F , Dobson CM. Protein misfolding, amyloid formation, and human disease: A summary of progress over the last decade. Annu Rev Biochem. 2017;86:27–68. 28498720 10.1146/annurev-biochem-061516-045115

[10] McAlary L , Plotkin SS, Yerbury JJ, Cashman NR. Prion-like propagation of protein misfolding and aggregation in amyotrophic lateral sclerosis. Front Mol Neurosci. 2019;12(262):262. 31736708 10.3389/fnmol.2019.00262 PMC6838634

[11] Polymenidou M , Cleveland DW. The seeds of neurodegeneration: Prion-like spreading in ALS. Cell. 2011;147(3):498–508. 22036560 10.1016/j.cell.2011.10.011 PMC3220614

[12] Bertolotti A . Importance of the subcellular location of protein deposits in neurodegenerative diseases. Curr Opin Neurobiol. 2018;51:127–133. 29631171 10.1016/j.conb.2018.03.004

[13] Taylor JP , Brown RH, Cleveland DW. Decoding ALS: From genes to mechanism. Nature. 2016;539(7628):197–206. 27830784 10.1038/nature20413 PMC5585017

[14] Shatunov A , Al-Chalabi A. The genetic architecture of ALS. Neurobiol Dis. 2021;147:105156. 33130222 10.1016/j.nbd.2020.105156

[15] Lattante S , Ciura S, Rouleau GA, Kabashi E. Defining the genetic connection linking amyotrophic lateral sclerosis (ALS) with frontotemporal dementia (FTD). Trends Genet. 2015;31(5):263–273. 25869998 10.1016/j.tig.2015.03.005

[16] Mathis S , Goizet C, Soulages A, Vallat JM, Masson GL. Genetics of amyotrophic lateral sclerosis: A review. J Neurol Sci. 2019;399:217–226. 30870681 10.1016/j.jns.2019.02.030

[17] Ling SC , Polymenidou M, Cleveland DW. Converging mechanisms in ALS and FTD: Disrupted RNA and protein homeostasis. Neuron. 2013;79(3):416–438. 23931993 10.1016/j.neuron.2013.07.033 PMC4411085

[18] Al-Chalabi A , Jones A, Troakes C, King A, Al-Sarraj S, van den Berg LH. The genetics and neuropathology of amyotrophic lateral sclerosis. Acta Neuropathol. 2012;124(3):339–352. 22903397 10.1007/s00401-012-1022-4

[19] Ciryam P , Tartaglia GG, Morimoto RI, Dobson CM, Vendruscolo M. Widespread aggregation and neurodegenerative diseases are associated with supersaturated proteins. Cell Rep. 2013;5(3):781–790. 24183671 10.1016/j.celrep.2013.09.043 PMC3883113

[20] Ciryam P , Kundra R, Morimoto RI, Dobson CM, Vendruscolo M. Supersaturation is a major driving force for protein aggregation in neurodegenerative diseases. Trends Pharmacol Sci. 2015;36(2):72–77. 25636813 10.1016/j.tips.2014.12.004 PMC4643722

[21] Nakashima-Yasuda H , Uryu K, Robinson J, et al Co-morbidity of TDP-43 proteinopathy in Lewy body related diseases. Acta Neuropathol. 2007;114(3):221–229. 17653732 10.1007/s00401-007-0261-2

[22] Amador-Ortiz C , Lin WL, Ahmed Z, et al TDP-43 immunoreactivity in hippocampal sclerosis and Alzheimer’s disease. Ann Neurol. 2007;61(5):435–445. 17469117 10.1002/ana.21154 PMC2677204

[23] Tziortzouda P , Van Den Bosch L, Hirth F. Triad of TDP43 control in neurodegeneration: Autoregulation, localization and aggregation. Nat Rev Neurosci. 2021;22(4):197–208. 33654312 10.1038/s41583-021-00431-1

[24] Wang Y , Branicky R, Noë A, Hekimi S. Superoxide dismutases: Dual roles in controlling ROS damage and regulating ROS signaling. J Cell Biol. 2018;217(6):1915–1928. 29669742 10.1083/jcb.201708007 PMC5987716

[25] Rosen DR . Mutations in Cu/Zn superoxide dismutase gene are associated with familial amyotrophic lateral sclerosis. Nature. 1993;364(6435):362. 8332197 10.1038/364362c0

[26] Reaume AG , Elliott JL, Hoffman EK, et al Motor neurons in Cu/Zn superoxide dismutase-deficient mice develop normally but exhibit enhanced cell death after axonal injury. Nat Genet. 1996;13(1):43–47. 8673102 10.1038/ng0596-43

[27] Fischer LR , Li Y, Asress SA, Jones DP, Glass JD. Absence of SOD1 leads to oxidative stress in peripheral nerve and causes a progressive distal motor axonopathy. Exp Neurol. 2012;233(1):163–171. 21963651 10.1016/j.expneurol.2011.09.020 PMC4068963

[28] Bruijn LI , Houseweart MK, Kato S, et al Aggregation and motor neuron toxicity of an ALS-linked SOD1 mutant independent from wild-type SOD1. Science. 1998;281(5384):1851–1854. 9743498 10.1126/science.281.5384.1851

[29] Bowling AC , Barkowski EE, McKenna-Yasek D, et al Superoxide dismutase concentration and activity in familial amyotrophic lateral sclerosis. J Neurochem. 1995;64(5):2366–2369. 7722523 10.1046/j.1471-4159.1995.64052366.x

[30] Cleveland DW , Laing N, Hurse PV, Brown RH. Toxic mutants in Charcot’s sclerosis. Nature. 1995;378(6555):342–343. 7477368 10.1038/378342a0

[31] Ayers JI , Diamond J, Sari A, et al Distinct conformers of transmissible misfolded SOD1 distinguish human SOD1-FALS from other forms of familial and sporadic ALS. Acta Neuropathol. 2016;132(6):827–840. 27704280 10.1007/s00401-016-1623-4 PMC5107152

[32] Paré B , Lehmann M, Beaudin M, et al Misfolded SOD1 pathology in sporadic amyotrophic lateral sclerosis. Sci Rep. 2018;8(1):14223. 30242181 10.1038/s41598-018-31773-z PMC6155098

[33] Forsberg K , Jonsson PA, Andersen PM, et al Novel antibodies reveal inclusions containing non-native SOD1 in sporadic ALS patients. PLoS One. 2010;5(7):e11552. 20644736 10.1371/journal.pone.0011552 PMC2904380

[34] Forsberg K , Graffmo K, Pakkenberg B, et al Misfolded SOD1 inclusions in patients with mutations in C9orf72 and other ALS/FTD-associated genes. J Neurol Neurosurg Psychiatry. 2019;90(8):861–869. 30992335 10.1136/jnnp-2018-319386 PMC6691870

[35] Pokrishevsky E , Grad LI, Yousefi M, Wang J, Mackenzie IR, Cashman NR. Aberrant localization of FUS and TDP43 is associated with misfolding of SOD1 in amyotrophic lateral sclerosis. PLoS One. 2012;7(4):e35050. 22493728 10.1371/journal.pone.0035050 PMC3320864

[36] Da Cruz S , Bui A, Saberi S, et al Misfolded SOD1 is not a primary component of sporadic ALS. Acta Neuropathol. 2017;134(1):97–111. 28247063 10.1007/s00401-017-1688-8 PMC5472502

[37] Ratti A , Buratti E. Physiological functions and pathobiology of TDP-43 and FUS/TLS proteins. J Neurochem. 2016;138:95–111. 27015757 10.1111/jnc.13625

[38] Panza F , Lozupone M, Seripa D, et al Development of disease-modifying drugs for frontotemporal dementia spectrum disorders. Nat Rev Neurol. 2020;16(4):213–228. 32203398 10.1038/s41582-020-0330-x

[39] Sreedharan J , Blair IP, Tripathi VB, et al TDP-43 mutations in familial and sporadic amyotrophic lateral sclerosis. Science. 2008;319(5870):1668–1672. 18309045 10.1126/science.1154584 PMC7116650

[40] Gasset-Rosa F , Lu S, Yu H, et al Cytoplasmic TDP-43 de-mixing independent of stress granules drives inhibition of nuclear import, loss of nuclear TDP-43, and cell death. Neuron. 2019;102(2):339–357.e7. 30853299 10.1016/j.neuron.2019.02.038 PMC6548321

[41] Purice MD , Taylor JP. Linking hnRNP function to ALS and FTD pathology. Front Neurosci. 2018;12:326. 29867335 10.3389/fnins.2018.00326 PMC5962818

[42] Arai T , Hasegawa M, Akiyama H, et al TDP-43 is a component of ubiquitin-positive tau-negative inclusions in frontotemporal lobar degeneration and amyotrophic lateral sclerosis. Biochem Biophys Res Commun. 2006;351(3):602–611. 17084815 10.1016/j.bbrc.2006.10.093

[43] Neumann M , Sampathu DM, Kwong LK, et al Ubiquitinated TDP-43 in frontotemporal lobar degeneration and amyotrophic lateral sclerosis. Science. 2006;314(5796):130–133. 17023659 10.1126/science.1134108

[44] Hasegawa M , Arai T, Nonaka T, et al Phosphorylated TDP-43 in frontotemporal lobar degeneration and amyotrophic lateral sclerosis. Ann Neurol. 2008;64(1):60–70. 18546284 10.1002/ana.21425 PMC2674108

[45] Kim G , Gautier O, Tassoni-Tsuchida E, Ma XR, Gitler AD. ALS genetics: Gains, losses, and implications for future therapies. Neuron. 2020;108(5):822–842. 32931756 10.1016/j.neuron.2020.08.022 PMC7736125

[46] Kwiatkowski TJ , Bosco DA, Leclerc AL, et al Mutations in the FUS/TLS gene on chromosome 16 cause familial amyotrophic lateral sclerosis. Science. 2009;323(5918):1205–1208. 19251627 10.1126/science.1166066

[47] Vance C , Rogelj B, Hortobágyi T, et al Mutations in FUS, an RNA processing protein, cause familial amyotrophic lateral sclerosis type 6. Science. 2009;323(5918):1208–1211. 19251628 10.1126/science.1165942 PMC4516382

[48] Tyzack GE , Luisier R, Taha DM, et al Widespread FUS mislocalization is a molecular hallmark of amyotrophic lateral sclerosis. Brain. 2019;142(9):2572–2580. 31368485 10.1093/brain/awz217 PMC6735815

[49] Korenkova O , Pepe A, Zurzolo C. Fine intercellular connections in development: TNTs, cytonemes, or intercellular bridges? Cell Stress. 2020;4(2):30–43. 32043076 10.15698/cst2020.02.212 PMC6997949

[50] Asai H , Ikezu S, Tsunoda S, et al Depletion of microglia and inhibition of exosome synthesis halt tau propagation. Nat Neurosci. 2015;18(11):1584–1593. 26436904 10.1038/nn.4132 PMC4694577

[51] Donnelly KM , DeLorenzo OR, Zaya AD, et al Phagocytic glia are obligatory intermediates in transmission of mutant huntingtin aggregates across neuronal synapses. Elife. 2020;9:e58499. 32463364 10.7554/eLife.58499 PMC7297539

[52] Wood H . Evidence for trans-synaptic and exo-synaptic tau propagation in Alzheimer disease. Nat Rev Neurol. 2015;11(12):665–665. 26526534 10.1038/nrneurol.2015.205

[53] Cicardi ME , Marrone L, Azzouz M, Trotti D. Proteostatic imbalance and protein spreading in amyotrophic lateral sclerosis. EMBO J. 2021;40(10):e106389. 33792056 10.15252/embj.2020106389 PMC8126909

[54] Hipp MS , Kasturi P, Hartl FU. The proteostasis network and its decline in ageing. Nat Rev Mol Cell Biol. 2019;20(7):421–435. 30733602 10.1038/s41580-019-0101-y

[55] Ruegsegger C , Saxena S. Proteostasis impairment in ALS. Brain Res. 2016;1648:571–579. 27033833 10.1016/j.brainres.2016.03.032

[56] Douglas PM , Dillin A. Protein homeostasis and aging in neurodegeneration. J Cell Biol. 2010;190(5):719–729. 20819932 10.1083/jcb.201005144 PMC2935559

[57] Hetz C . Adapting the proteostasis capacity to sustain brain healthspan. Cell. 2021;184(6):1545–1560. 33691137 10.1016/j.cell.2021.02.007

[58] Peng C , Trojanowski JQ, Lee VMY. Protein transmission in neurodegenerative disease. Nat Rev Neurol. 2020;16(4):199–212. 32203399 10.1038/s41582-020-0333-7 PMC9242841

[59] Victoria GS , Zurzolo C. The spread of prion-like proteins by lysosomes and tunneling nanotubes: Implications for neurodegenerative diseases. J Cell Biol. 2017;216(9):2633–2644. 28724527 10.1083/jcb.201701047 PMC5584166

[60] Vilette D , Courte J, Peyrin JM, et al Cellular mechanisms responsible for cell-to-cell spreading of prions. Cell Mol Life Sci. 2018;75(14):2557–2574. 29761205 10.1007/s00018-018-2823-y PMC11105574

[61] Gallotta I , Sandhu A, Peters M, et al Extracellular proteostasis prevents aggregation during pathogenic attack. Nature. 2020;584(7821):410–414. 32641833 10.1038/s41586-020-2461-z

[62] Melentijevic I , Toth ML, Arnold ML, et al *C. elegans* neurons jettison protein aggregates and mitochondria under neurotoxic stress. Nature. 2017;542(7641):367–371. 28178240 10.1038/nature21362 PMC5336134

[63] Rabouille C . Pathways of unconventional protein secretion. Trends Cell Biol. 2017;27(3):230–240. 27989656 10.1016/j.tcb.2016.11.007

[64] Sitia R , Rubartelli A. Evolution, role in inflammation, and redox control of leaderless secretory proteins. J Biol Chem. 2020;295(22):7799–7811. 32332096 10.1074/jbc.REV119.008907 PMC7261794

[65] Volkmar N , Fenech E, Christianson JC. New MAPS for misfolded proteins. Nat Cell Biol. 2016;18(7):724–726. 27350445 10.1038/ncb3381

[66] Shiina Y , Arima K, Tabunoki H, Satoh JI. TDP-43 dimerizes in human cells in culture. Cell Mol Neurobiol. 2010;30(4):641–652. 20043239 10.1007/s10571-009-9489-9 PMC11498789

[67] Holm MM , Kaiser J, Schwab ME. Extracellular vesicles: Multimodal envoys in neural maintenance and repair. Trends Neurosci. 2018;41(6):360–372. 29605090 10.1016/j.tins.2018.03.006

[68] Lim YJ . Lee SJ. Are exosomes the vehicle for protein aggregate propagation in neurodegenerative diseases? Acta Neuropathol Commun. 2017;5(1):64. 28851422 10.1186/s40478-017-0467-z PMC5576311

[69] Nickel W . The mystery of nonclassical protein secretion. A current view on cargo proteins and potential export routes. Eur J Biochem. 2003;270(10):2109–2119. 12752430 10.1046/j.1432-1033.2003.03577.x

[70] Pallotta MT , Nickel W. FGF2 and IL-1β—explorers of unconventional secretory pathways at a glance. J Cell Sci. 2020;133(21):jcs250449. 33154173 10.1242/jcs.250449

[71] Cruz-Garcia D , Malhotra V, Curwin AJ. Unconventional protein secretion triggered by nutrient starvation. Semin Cell Dev Biol. 2018;83:22–28. 29486236 10.1016/j.semcdb.2018.02.021

[72] Urushitani M , Sik A, Sakurai T, Nukina N, Takahashi R, Julien JP. Chromogranin-mediated secretion of mutant superoxide dismutase proteins linked to amyotrophic lateral sclerosis. Nat Neurosci. 2006;9(1):108–118. 16369483 10.1038/nn1603

[73] Leidal AM , Huang HH, Marsh T, et al The LC3-conjugation machinery specifies the loading of RNA-binding proteins into extracellular vesicles. Nat Cell Biol. 2020;22(2):187–199. 31932738 10.1038/s41556-019-0450-y PMC7007875

[74] Santillo M , Secondo A, Serù R, et al Evidence of calcium- and SNARE-dependent release of CuZn superoxide dismutase from rat pituitary GH3 cells and synaptosomes in response to depolarization. J Neurochem. 2007;102(3):679–685. 17403136 10.1111/j.1471-4159.2007.04538.x

[75] Pluthero FG , Shreeve M, Eskinazi D, et al Purification of an inhibitor of erythroid progenitor cell cycling and antagonist to interleukin 3 from mouse marrow cell supernatants and its identification as cytosolic superoxide dismutase. J Cell Biol. 1990;111(3):1217–1223. 2391363 10.1083/jcb.111.3.1217 PMC2116302

[76] Cimini V , Ruggiero G, Buonomo T, et al CuZn-superoxide dismutase in human thymus: Immunocytochemical localisation and secretion in thymus-derived epithelial and fibroblast cell lines. Histochem Cell Biol. 2002;118(2):163–169. 12189519 10.1007/s00418-002-0429-8

[77] Cruz-Garcia D , Brouwers N, Duran JM, Mora G, Curwin AJ, Malhotra V. A diacidic motif determines unconventional secretion of wild-type and ALS-linked mutant SOD1. J Cell Biol. 2017;216(9):2691–2700. 28794127 10.1083/jcb.201704056 PMC5584182

[78] Mondola P , Annella T, Santillo M, Santangelo F. Evidence for secretion of cytosolic CuZn superoxide dismutase by Hep G2 cells and human fibroblasts. Int J Biochem Cell Biol. 1996;28(6):677–681. 8673732 10.1016/1357-2725(96)00004-0

[79] Mondola P , Ruggiero G, Serù R, et al The Cu, Zn superoxide dismutase in neuroblastoma SK-N-BE cells is exported by a microvesicles dependent pathway. Brain Res Mol Brain Res. 2003;110(1):45–51. 12573532 10.1016/s0169-328x(02)00583-1

[80] Yu L , Chen Y, Tooze SA. Autophagy pathway: Cellular and molecular mechanisms. Autophagy. 2018;14(2):207–215. 28933638 10.1080/15548627.2017.1378838 PMC5902171

[81] Andersen PM , Nilsson P, Keränen ML, et al Phenotypic heterogeneity in motor neuron disease patients with CuZn-superoxide dismutase mutations in Scandinavia. Brain. 1997;120(10):1723–1737. 9365366 10.1093/brain/120.10.1723

[82] Segovia-Silvestre T , Andreu AL, Vives-Bauza C, Garcia-Arumi E, Cervera C, Gamez J. A novel exon 3 mutation (D76V) in the SOD1 gene associated with slowly progressive ALS. Amyotroph Lateral Scler Other Motor Neuron Disord. 2002;3(2):69–74. 12215228 10.1080/146608202760196039

[83] Gomes C , Keller S, Altevogt P, Costa J. Evidence for secretion of Cu, Zn superoxide dismutase via exosomes from a cell model of amyotrophic lateral sclerosis. Neurosci Lett. 2007;428(1):43–46. 17942226 10.1016/j.neulet.2007.09.024

[84] Basso M , Pozzi S, Tortarolo M, et al Mutant copper-zinc superoxide dismutase (SOD1) induces protein secretion pathway alterations and exosome release in astrocytes: Implications for disease spreading and motor neuron pathology in amyotrophic lateral sclerosis. J Biol Chem. 2013;288(22):15699–15711. 23592792 10.1074/jbc.M112.425066 PMC3668729

[85] Grad LI , Yerbury JJ, Turner BJ, et al Intercellular propagated misfolding of wild-type Cu/Zn superoxide dismutase occurs via exosome-dependent and -independent mechanisms. Proc Natl Acad Sci U S A. 2014;111(9):3620–3625. 24550511 10.1073/pnas.1312245111 PMC3948312

[86] Petrozziello T , Secondo A, Tedeschi V, et al ApoSOD1 lacking dismutase activity neuroprotects motor neurons exposed to beta-methylamino-L-alanine through the Ca2+/Akt/ERK1/2 prosurvival pathway. Cell Death Differ. 2017;24(3):511–522. 28085149 10.1038/cdd.2016.154 PMC5344211

[87] Cruz-Garcia D , Brouwers N, Malhotra V, Curwin AJ. Reactive oxygen species triggers unconventional secretion of antioxidants and Acb1. J Cell Biol. 2020;219(4). 10.1083/jcb.201905028 PMC7147093 32328640

[88] Smith HL , Freeman OJ, Butcher AJ, et al Astrocyte unfolded protein response induces a specific reactivity state that causes non-cell-autonomous neuronal degeneration. Neuron. 2020;105(5):855–866.e5. 31924446 10.1016/j.neuron.2019.12.014 PMC7054837

[89] Urushitani M , Ezzi SA, Matsuo A, Tooyama I, Julien JP. The endoplasmic reticulum–Golgi pathway is a target for translocation and aggregation of mutant superoxide dismutase linked to ALS. FASEB J. 2008;22(7):2476–2487. 18337461 10.1096/fj.07-092783

[90] Chang RC , Parakh S, Coates JR, Long S, Atkin JD. Protein disulphide isomerase is associated with mutant SOD1 in canine degenerative myelopathy. Neuroreport. 2019;30(1):8–13. 30422940 10.1097/WNR.0000000000001151

[91] Medinas DB , Rozas P, Martínez Traub F, et al Endoplasmic reticulum stress leads to accumulation of wild-type SOD1 aggregates associated with sporadic amyotrophic lateral sclerosis. Proc Natl Acad Sci U S A. 2018;115(32):8209–8214. 30038021 10.1073/pnas.1801109115 PMC6094144

[92] Allen WJ , Collinson I, Römisch K. Post-translational protein transport by the Sec complex. Trends Biochem Sci. 2019;44(6):481–483. 30962027 10.1016/j.tibs.2019.03.003

[93] Haßdenteufel S , Nguyen D, Helms V, Lang S, Zimmermann R. ER import of small human presecretory proteins: Components and mechanisms. FEBS Lett. 2019;593(18):2506–2524. 31325177 10.1002/1873-3468.13542

[94] Xu Y , Cui L, Dibello A, et al DNAJC5 facilitates USP19-dependent unconventional secretion of misfolded cytosolic proteins. Cell Discov. 2018;4(11):11. 29531792 10.1038/s41421-018-0012-7 PMC5838229

[95] Lee JG , Takahama S, Zhang G, Tomarev SI, Ye Y. Unconventional secretion of misfolded proteins promotes adaptation to proteasome dysfunction in mammalian cells. Nat Cell Biol. 2016;18(7):765–776. 27295555 10.1038/ncb3372 PMC10701763

[96] Thomas EV , Fenton WA, McGrath J, Horwich AL. Transfer of pathogenic and nonpathogenic cytosolic proteins between spinal cord motor neurons in vivo in chimeric mice. Proc Natl Acad Sci U S A. 2017;114(15):E3139–E3148. 28348221 10.1073/pnas.1701465114 PMC5393223

[97] Rudnick ND , Griffey CJ, Guarnieri P, et al Distinct roles for motor neuron autophagy early and late in the SOD1G93A mouse model of ALS. Proc Natl Acad Sci U S A. 2017;114(39):E8294–E8303. 28904095 10.1073/pnas.1704294114 PMC5625902

[98] Ponpuak M , Mandell MA, Kimura T, Chauhan S, Cleyrat C, Deretic V. Secretory autophagy. Curr Opin Cell Biol. 2015;35:106–116. 25988755 10.1016/j.ceb.2015.04.016 PMC4529791

[99] Nilsson P , Loganathan K, Sekiguchi M, et al Aβ secretion and plaque formation depend on autophagy. Cell Rep. 2013;5(1):61–69. 24095740 10.1016/j.celrep.2013.08.042

[100] Feiler MS , Strobel B, Freischmidt A, et al TDP-43 is intercellularly transmitted across axon terminals. J Cell Biol. 2015;211(4):897–911. 26598621 10.1083/jcb.201504057 PMC4657165

[101] Ishii T , Kawakami E, Endo K, Misawa H, Watabe K. Formation and spreading of TDP-43 aggregates in cultured neuronal and glial cells demonstrated by time-lapse imaging. PLoS One. 2017;12(6):e0179375. 28599005 10.1371/journal.pone.0179375 PMC5466347

[102] Iguchi Y , Eid L, Parent M, et al Exosome secretion is a key pathway for clearance of pathological TDP-43. Brain. 2016;139:3187–3201. 27679482 10.1093/brain/aww237 PMC5840881

[103] Nonaka T , Masuda-Suzukake M, Arai T, et al Prion-like properties of pathological TDP-43 aggregates from diseased brains. Cell Rep. 2013;4(1):124–134. 23831027 10.1016/j.celrep.2013.06.007

[104] Sproviero D , La Salvia S, Giannini M, et al Pathological proteins are transported by extracellular vesicles of sporadic amyotrophic lateral sclerosis patients. Front Neurosci. 2018;12:487. 30072868 10.3389/fnins.2018.00487 PMC6060258

[105] Delorme-Axford E , Klionsky DJ. The LC3-conjugation machinery specifies cargo loading and secretion of extracellular vesicles. Autophagy. 2020;16:1169–1171. 32401566 10.1080/15548627.2020.1760057 PMC7469661

[106] Münch C , O’Brien J, Bertolotti A. Prion-like propagation of mutant superoxide dismutase-1 misfolding in neuronal cells. Proc Natl Acad Sci U S A. 2011;108(9):3548–3553. 21321227 10.1073/pnas.1017275108 PMC3048161

[107] Grad LI , Pokrishevsky E, Silverman JM, Cashman NR. Exosome-dependent and independent mechanisms are involved in prion-like transmission of propagated Cu/Zn superoxide dismutase misfolding. Prion. 2014;8(5):331–335. 25551548 10.4161/19336896.2014.983398 PMC4601269

[108] Mercer J , Helenius A. Gulping rather than sipping: Macropinocytosis as a way of virus entry. Curr Opin Microbiol. 2012;15(4):490–499. 22749376 10.1016/j.mib.2012.05.016

[109] Swanson JA . Shaping cups into phagosomes and macropinosomes. Nat Rev Mol Cell Biol. 2008;9(8):639–649. 18612320 10.1038/nrm2447 PMC2851551

[110] Zeineddine R , Pundavela JF, Corcoran L, et al SOD1 protein aggregates stimulate macropinocytosis in neurons to facilitate their propagation. Mol Neurodegener. 2015;10:57. 26520394 10.1186/s13024-015-0053-4 PMC4628302

[111] Zhong Z , Grasso L, Sibilla C, Stevens TJ, Barry N, Bertolotti A. Prion-like protein aggregates exploit the RHO GTPase to cofilin-1 signaling pathway to enter cells. EMBO J. 2018;37(6):e97822. 10.15252/embj.201797822 PMC5852416 29496740

[112] Yerbury JJ . Protein aggregates stimulate macropinocytosis facilitating their propagation. Prion. 2016;10(2):119–126. 26963158 10.1080/19336896.2016.1141860 PMC4981200

[113] Zhao W , Beers DR, Henkel JS, et al Extracellular mutant SOD1 induces microglial-mediated motoneuron injury. Glia. 2010;58(2):231–243. 19672969 10.1002/glia.20919 PMC2784168

[114] Roberts K , Zeineddine R, Corcoran L, Li W, Campbell IL, Yerbury JJ. Extracellular aggregated Cu/Zn superoxide dismutase activates microglia to give a cytotoxic phenotype. Glia. 2013;61(3):409–419. 23281114 10.1002/glia.22444

[115] Loughlin FE , Wilce JA. TDP-43 and FUS-structural insights into RNA recognition and self-association. Curr Opin Struct Biol. 2019;59:134–142. 31479821 10.1016/j.sbi.2019.07.012

[116] Mompean M , Laurents DV. Intrinsically disordered domains, amyloids and protein liquid phases: Evolving concepts and open questions. Protein Pept Lett. 2017;24(4):281–293. 28176659 10.2174/0929866524666170206122106

[117] Deng HX , Shi Y, Furukawa Y, et al Conversion to the amyotrophic lateral sclerosis phenotype is associated with intermolecular linked insoluble aggregates of SOD1 in mitochondria. Proc Natl Acad Sci U S A. 2006;103(18):7142–7147. 16636275 10.1073/pnas.0602046103 PMC1447523

[118] Prudencio M , Durazo A, Whitelegge JP, Borchelt DR. An examination of wild-type SOD1 in modulating the toxicity and aggregation of ALS-associated mutant SOD1. Hum Mol Genet. 2010;19(24):4774–4789. 20871097 10.1093/hmg/ddq408 PMC2989888

[119] Wang L , Deng HX, Grisotti G, Zhai H, Siddique T, Roos RP. Wild-type SOD1 overexpression accelerates disease onset of a G85R SOD1 mouse. Hum Mol Genet. 2009;18(9):1642–1651. 19233858 10.1093/hmg/ddp085 PMC2667291

[120] Han-Xiang D , Hujun J, Ronggen F, et al Molecular dissection of ALS-associated toxicity of SOD1 in transgenic mice using an exon-fusion approach. Hum Mol Genet. 2008;17(15):2310–2319. 18424447 10.1093/hmg/ddn131 PMC2465800

[121] Witan H , Kern A, Koziollek-Drechsler I, Wade R, Behl C, Clement AM. Heterodimer formation of wild-type and amyotrophic lateral sclerosis-causing mutant Cu/Zn-superoxide dismutase induces toxicity independent of protein aggregation. Hum Mol Genet. 2008;17(10):1373–1385. 18211954 10.1093/hmg/ddn025

[122] Witan H , Gorlovoy P, Kaya AM, et al Wild-type Cu/Zn superoxide dismutase (SOD1) does not facilitate, but impedes the formation of protein aggregates of amyotrophic lateral sclerosis causing mutant SOD1. Neurobiol Dis. 2009;36(2):331–342. 19660548 10.1016/j.nbd.2009.07.024

[123] Banci L , Bertini I, Durazo A, et al Metal-free superoxide dismutase forms soluble oligomers under physiological conditions: A possible general mechanism for familial ALS. Proc Natl Acad Sci U S A. 2007;104(27):11263–11267. 17592131 10.1073/pnas.0704307104 PMC1899188

[124] Banci L , Bertini I, Boca M, et al SOD1 and amyotrophic lateral sclerosis: Mutations and oligomerization. PLoS One. 2008;3(2):e1677. 18301754 10.1371/journal.pone.0001677 PMC2250751

[125] Chan PK , Chattopadhyay M, Sharma S, et al Structural similarity of wild-type and ALS-mutant superoxide dismutase-1 fibrils using limited proteolysis and atomic force microscopy. Proc Natl Acad Sci U S A. 2013;110(27):10934–10939. 23781106 10.1073/pnas.1309613110 PMC3704032

[126] Chattopadhyay M , Valentine JS. Aggregation of copper-zinc superoxide dismutase in familial and sporadic ALS. Antioxid Redox Signal. 2009;11(7):1603–1614. 19271992 10.1089/ars.2009.2536 PMC2842589

[127] Chia R , Tattum MH, Jones S, Collinge J, Fisher EMC, Jackson GS. Superoxide dismutase 1 and tgSOD1 mouse spinal cord seed fibrils, suggesting a propagative cell death mechanism in amyotrophic lateral sclerosis. PLoS One. 2010;5(5):e10627. 20498711 10.1371/journal.pone.0010627 PMC2869360

[128] Furukawa Y , Kaneko K, Yamanaka K, O’Halloran TV, Nukina N. Complete loss of post-translational modifications triggers fibrillar aggregation of SOD1 in the familial form of amyotrophic lateral sclerosis. J Biol Chem. 2008;283(35):24167–24176. 18552350 10.1074/jbc.M802083200 PMC3259764

[129] Grad LI , Guest WC, Yanai A, et al Intermolecular transmission of superoxide dismutase 1 misfolding in living cells. Proc Natl Acad Sci U S A. 2011;108(39):16398–16403. 21930926 10.1073/pnas.1102645108 PMC3182705

[130] Banci L , Bertini I, Cramaro F, Del Conte R, Viezzoli MS. Solution structure of Apo Cu, Zn superoxide dismutase: Role of metal ions in protein folding. Biochemistry. 2003;42(32):9543–9553. 12911296 10.1021/bi034324m

[131] Cohen NR , Kayatekin C, Zitzewitz JA, Bilsel O, Matthews CR. Friction-limited folding of disulfide-reduced monomeric SOD1. Biophys J. 2020;118(8):1992–2000. 32191862 10.1016/j.bpj.2020.02.028 PMC7175461

[132] Ding F , Furukawa Y, Nukina N, Dokholyan NV. Local unfolding of Cu, Zn superoxide dismutase monomer determines the morphology of fibrillar aggregates. J Mol Biol. 2012;421(4–5):548–560. 22210350 10.1016/j.jmb.2011.12.029 PMC3320695

[133] Lindberg MJ , Normark J, Holmgren A, Oliveberg M. Folding of human superoxide dismutase: Disulfide reduction prevents dimerization and produces marginally stable monomers. Proc Natl Acad Sci U S A. 2004;101(45):15893–15898. 15522970 10.1073/pnas.0403979101 PMC528748

[134] Mojumdar S S , Scholl Z N, Dee DR, et al Partially native intermediates mediate misfolding of SOD1 in single-molecule folding trajectories. Nat Commun. 2017;8(1):1881. 29192167 10.1038/s41467-017-01996-1 PMC5709426

[135] Kayatekin C , Zitzewitz JA, Matthews CR. Disulfide-reduced ALS Variants of Cu, Zn superoxide dismutase exhibit increased populations of unfolded species. J Mol Biol. 2010;398(2):320–331. 20184893 10.1016/j.jmb.2010.02.034 PMC3075925

[136] Ivanova MI , Sievers SA, Guenther EL, et al Aggregation-triggering segments of SOD1 fibril formation support a common pathway for familial and sporadic ALS. Proc Natl Acad Sci U S A. 2014;111(1):197–201. 24344300 10.1073/pnas.1320786110 PMC3890817

[137] Sangwan S , Zhao A, Adams KL, et al Atomic structure of a toxic, oligomeric segment of SOD1 linked to amyotrophic lateral sclerosis (ALS). Proc Natl Acad Sci U S A. 2017;114(33):8770–8775. 28760994 10.1073/pnas.1705091114 PMC5565441

[138] Sangwan S , Sawaya MR, Murray KA, Hughes MP, Eisenberg DS. Atomic structures of corkscrew-forming segments of SOD1 reveal varied oligomer conformations. Protein Sci. 2018;27(7):1231–1242. 29453800 10.1002/pro.3391 PMC6032342

[139] Furukawa Y , Fu R, Deng HX, Siddique T, O’Halloran TV. Disulfide cross-linked protein represents a significant fraction of ALS-associated Cu, Zn-superoxide dismutase aggregates in spinal cords of model mice. Proc Natl Acad Sci U S A. 2006;103(18):7148–7153. 16636274 10.1073/pnas.0602048103 PMC1447524

[140] Karch CM , Borchelt DR. A limited role for disulfide cross-linking in the aggregation of mutant SOD1 linked to familial amyotrophic lateral sclerosis. J Biol Chem. 2008;283(20):13528–13537. 18316367 10.1074/jbc.M800564200 PMC2376231

[141] Grad LI , Pokrishevsky E, Cashman NR. Intercellular prion-like conversion and transmission of Cu/Zn superoxide dismutase (SOD1) in cell culture. Methods Mol Biol. 2017;1658:357–367. 28861801 10.1007/978-1-4939-7244-9_24

[142] Afroz T , Hock EM, Ernst P, et al Functional and dynamic polymerization of the ALS-linked protein TDP-43 antagonizes its pathologic aggregation. Nat Commun. 2017;8(1):45. 28663553 10.1038/s41467-017-00062-0 PMC5491494

[143] Hallegger M , Chakrabarti AM, Lee FCY, et al TDP-43 condensation properties specify its RNA-binding and regulatory repertoire. Cell. 2021;184(18):4680–4696.e22. 34380047 10.1016/j.cell.2021.07.018 PMC8445024

[144] Mann JR , Donnelly CJ. RNA modulates physiological and neuropathological protein phase transitions. Neuron. 2021;109(17):2663–2681. 34297914 10.1016/j.neuron.2021.06.023 PMC8434763

[145] Maharana S , Wang J, Papadopoulos DK, et al RNA buffers the phase separation behavior of prion-like RNA binding proteins. Science. 2018;360(6391):918–921. 29650702 10.1126/science.aar7366 PMC6091854

[146] Mann JR , Gleixner AM, Mauna JC, et al RNA binding antagonizes neurotoxic phase transitions of TDP-43. Neuron. 2019;102(2):321–338.e8. 30826182 10.1016/j.neuron.2019.01.048 PMC6472983

[147] French RL , Grese ZR, Aligireddy H, et al Detection of TAR DNA-binding protein 43 (TDP-43) oligomers as initial intermediate species during aggregate formation. J Biol Chem. 2019;294(17):6696–6709. 30824544 10.1074/jbc.RA118.005889 PMC6497947

[148] Ling SC , Albuquerque CP, Han JS, et al ALS-associated mutations in TDP-43 increase its stability and promote TDP-43 complexes with FUS/TLS. Proc Natl Acad Sci U S A. 2010;107(30):13318–13323. 20624952 10.1073/pnas.1008227107 PMC2922163

[149] Hergesheimer RC , Chami AA, de Assis DR, et al The debated toxic role of aggregated TDP-43 in amyotrophic lateral sclerosis: A resolution in sight? Brain. 2019;142(5):1176–1194. 30938443 10.1093/brain/awz078 PMC6487324

[150] Loganathan S , Lehmkuhl EM, Eck RJ, Zarnescu DC. To be or not to be…toxic—is RNA association with TDP-43 complexes deleterious or protective in neurodegeneration? Front Mol Biosci. 2019;6:154. 31998750 10.3389/fmolb.2019.00154 PMC6965497

[151] Cutler AA , Ewachiw TE, Corbet GA, Parker R, Olwin BB. Myo-granules connect physiology and pathophysiology. J Exp Neurosci. 2019;13:117906951984215. 10.1177/1179069519842157 PMC6463236 31019368

[152] Vogler TO , Wheeler JR, Nguyen ED, et al TDP-43 and RNA form amyloid-like myo-granules in regenerating muscle. Nature. 2018;563(7732):508–513. 30464263 10.1038/s41586-018-0665-2 PMC6324568

[153] Dewey CM , Cenik B, Sephton CF, et al TDP-43 is directed to stress granules by sorbitol, a novel physiological osmotic and oxidative stressor. Mol Cell Biol. 2011;31(5):1098–1108. 21173160 10.1128/MCB.01279-10 PMC3067820

[154] Mateju D , Franzmann TM, Patel A, et al An aberrant phase transition of stress granules triggered by misfolded protein and prevented by chaperone function. EMBO J. 2017;36(12):1669–1687. 28377462 10.15252/embj.201695957 PMC5470046

[155] Walker AK , Soo KY, Sundaramoorthy V, et al ALS-associated TDP-43 induces endoplasmic reticulum stress, which drives cytoplasmic TDP-43 accumulation and stress granule formation. PLoS One. 2013;8(11):e81170. 24312274 10.1371/journal.pone.0081170 PMC3843686

[156] Zhu L , Xu M, Yang M, et al An ALS-mutant TDP-43 neurotoxic peptide adopts an anti-parallel β-structure and induces TDP-43 redistribution. Hum Mol Genet. 2014;23(25):6863–6877. 25113748 10.1093/hmg/ddu409 PMC4245047

[157] Bogaert E , Boeynaems S, Kato M, et al Molecular dissection of FUS points at synergistic effect of low-complexity domains in toxicity. Cell Rep. 2018;24(3):529–537.e4. 30021151 10.1016/j.celrep.2018.06.070 PMC6077250

[158] Holehouse AS , Pappu RV. FUS zigzags its way to cross beta. Cell. 2017;171(3):499–500. 29053965 10.1016/j.cell.2017.10.007

[159] Patel A , Lee HO, Jawerth L, et al A liquid-to-solid phase transition of the ALS protein FUS accelerated by disease mutation. Cell. 2015;162(5):1066–1077. 26317470 10.1016/j.cell.2015.07.047

[160] Murray DT , Kato M, Lin Y, et al Structure of FUS protein fibrils and its relevance to self-assembly and phase separation of low-complexity domains. Cell. 2017;171(3):615–627.e16. 28942918 10.1016/j.cell.2017.08.048 PMC5650524

[161] Murthy AC , Dignon GL, Kan Y, et al Molecular interactions underlying liquid–liquid phase separation of the FUS low-complexity domain. Nat Struct Mol Biol. 2019;26(7):637–648. 31270472 10.1038/s41594-019-0250-x PMC6613800

[162] Niaki AG , Sarkar J, Cai X, et al Loss of dynamic RNA interaction and aberrant phase separation induced by two distinct types of ALS/FTD-linked FUS mutations. Mol Cell. 2020;77(1):82–94.e4. 31630970 10.1016/j.molcel.2019.09.022 PMC6943187

[163] Murakami T , Qamar S, Lin JQ, et al ALS/FTD mutation-induced phase transition of FUS liquid droplets and reversible hydrogels into irreversible hydrogels impairs RNP granule function. Neuron. 2015;88(4):678–690. 26526393 10.1016/j.neuron.2015.10.030 PMC4660210

[164] Ayers JI , Fromholt S, Koch M, et al Experimental transmissibility of mutant SOD1 motor neuron disease. Acta Neuropathol. 2014;128(6):791–803. 25262000 10.1007/s00401-014-1342-7

[165] Bergh J , Zetterström P, Andersen PM, et al Structural and kinetic analysis of protein-aggregate strains in vivo using binary epitope mapping. Proc Natl Acad Sci U S A. 2015;112(14):4489–4494. 25802384 10.1073/pnas.1419228112 PMC4394267

[166] Bidhendi EE , Bergh J, Zetterström P, Andersen PM, Marklund SL, Brännström T. Two superoxide dismutase prion strains transmit amyotrophic lateral sclerosis-like disease. J Clin Invest. 2016;126(6):2249–2253. 27140399 10.1172/JCI84360 PMC4887173

[167] Bidhendi E E , Bergh J, Zetterström P, et al Mutant superoxide dismutase aggregates from human spinal cord transmit amyotrophic lateral sclerosis. Acta Neuropathol. 2018;136(6):939–953. 30284034 10.1007/s00401-018-1915-y PMC6280858

[168] Keskin I , Ekhtiari Bidhendi E, Marklund M, et al Peripheral administration of SOD1 aggregates does not transmit pathogenic aggregation to the CNS of SOD1 transgenic mice. Acta Neuropathol Commun. 2021;9(1):111. 34158126 10.1186/s40478-021-01211-9 PMC8220797

[169] Ayers JI , Xu G, Dillon K, et al Variation in the vulnerability of mice expressing human superoxide dismutase 1 to prion-like seeding: A study of the influence of primary amino acid sequence. Acta Neuropathol Commun. 2021;9(1):92. 34016165 10.1186/s40478-021-01191-w PMC8139116

[170] Porta S , Xu Y, Restrepo CR, et al Patient-derived frontotemporal lobar degeneration brain extracts induce formation and spreading of TDP-43 pathology in vivo. Nat Commun. 2018;9(1):4220. 30310141 10.1038/s41467-018-06548-9 PMC6181940

[171] Mishra PS , Boutej H, Soucy G, et al Transmission of ALS pathogenesis by the cerebrospinal fluid. Acta Neuropathol Commun. 2020;8(1):65. 32381112 10.1186/s40478-020-00943-4 PMC7206749

[172] Ding X , Xiang Z, Qin C, et al Spreading of TDP-43 pathology via pyramidal tract induces ALS-like phenotypes in TDP-43 transgenic mice. Acta Neuropathol Commun. 2021;9(1):15. 33461623 10.1186/s40478-020-01112-3 PMC7814549

[173] Zhang R , Chen Y, Wang X, et al Spreading of pathological TDP-43 along corticospinal tract axons induces ALS-like phenotypes in Atg5+/− mice. Int J Biol Sci. 2021;17(2):390–401. 33613100 10.7150/ijbs.53872 PMC7893595

[174] Braak H , Brettschneider J, Ludolph AC, Lee VM, Trojanowski JQ, Del Tredici K. Amyotrophic lateral sclerosis—a model of corticofugal axonal spread. Nat Rev Neurol. 2013;9(12):708–714. 24217521 10.1038/nrneurol.2013.221 PMC3943211

[175] Benson MD , Buxbaum JN, Eisenberg DS, et al Amyloid nomenclature 2018: Recommendations by the International Society of Amyloidosis (ISA) nomenclature committee. Amyloid. 2018;25(4):215–219. 30614283 10.1080/13506129.2018.1549825

[176] Purro SA , Farrow MA, Linehan J, et al Transmission of amyloid-β protein pathology from cadaveric pituitary growth hormone. Nature. 2018;564(7736):415–419. 30546139 10.1038/s41586-018-0790-y PMC6708408

[177] Brown P , Gibbs CJ, Rodgers-Johnson P, et al Human spongiform encephalopathy: The National Institutes of Health series of 300 cases of experimentally transmitted disease. Ann Neurol. 1994;35(5):513–529. 8179297 10.1002/ana.410350504

[178] Lam S , Petit F, Hérard AS, et al Transmission of amyloid-beta and tau pathologies is associated with cognitive impairments in a primate. Acta Neuropathol Commun. 2021;9:165. 34641980 10.1186/s40478-021-01266-8 PMC8507137

[179] Scekic-Zahirovic J , Fischer M, Stuart-Lopez G, et al Evidence that corticofugal propagation of ALS pathology is not mediated by prion-like mechanism. Prog Neurobiol. 2021;200:101972. 33309802 10.1016/j.pneurobio.2020.101972

[180] Brooks BR . The role of axonal transport in neurodegenerative disease spread: A meta-analysis of experimental and clinical poliomyelitis compares with amyotrophic lateral sclerosis. Can J Neurol Sci. 1991;18(S3):435–438. 1718581 10.1017/s0317167100032625

[181] Ayers JI , Fromholt SE, O’Neal VM, Diamond JH, Borchelt DR. Prion-like propagation of mutant SOD1 misfolding and motor neuron disease spread along neuroanatomical pathways. Acta Neuropathol. 2016;131(1):103–114. 26650262 10.1007/s00401-015-1514-0 PMC4699876

[182] Boillée S , Yamanaka K, Lobsiger CS, et al Onset and progression in inherited ALS determined by motor neurons and microglia. Science. 2006;312(5778):1389–1392. 16741123 10.1126/science.1123511

[183] Gros-Louis F , Soucy G, Larivière R, Julien JP. Intracerebroventricular infusion of monoclonal antibody or its derived Fab fragment against misfolded forms of SOD1 mutant delays mortality in a mouse model of ALS. J Neurochem. 2010;113(5):1188–1199. 20345765 10.1111/j.1471-4159.2010.06683.x

[184] Patel P , Kriz J, Gravel M, et al Adeno-associated virus-mediated delivery of a recombinant single-chain antibody against misfolded superoxide dismutase for treatment of amyotrophic lateral sclerosis. Mol Ther. 2014;22(3):498–510. 24394188 10.1038/mt.2013.239 PMC3944333

[185] Maier M , Welt T, Wirth F, et al A human-derived antibody targets misfolded SOD1 and ameliorates motor symptoms in mouse models of amyotrophic lateral sclerosis. Sci Transl Med. 2018;10(470):eaah3924. 30518612 10.1126/scitranslmed.aah3924

[186] Urushitani M , Ezzi SA, Julien JP. Therapeutic effects of immunization with mutant superoxide dismutase in mice models of amyotrophic lateral sclerosis. Proc Natl Acad Sci U S A. 2007;104(7):2495–2500. 17277077 10.1073/pnas.0606201104 PMC1790867

[187] Zhao B , Marciniuk K, Gibbs E, Yousefi M, Napper S, Cashman NR. Therapeutic vaccines for amyotrophic lateral sclerosis directed against disease specific epitopes of superoxide dismutase 1. Vaccine. 2019;37(35):4920–4927. 31324499 10.1016/j.vaccine.2019.07.044

[188] Liu AYC , Mathur R, Mei N, Langhammer CG, Babiarz B, Firestein BL. Neuroprotective drug riluzole amplifies the heat shock factor 1 (HSF1)- and glutamate transporter 1 (GLT1)-dependent cytoprotective mechanisms for neuronal survival. J Biol Chem. 2011;286(4):2785–2794. 21098017 10.1074/jbc.M110.158220 PMC3024774

[189] Kalmar B , Greensmith L. Cellular chaperones as therapeutic targets in ALS to restore protein homeostasis and improve cellular function. Front Mol Neurosci. 2017;10:251. 28943839 10.3389/fnmol.2017.00251 PMC5596081

[190] Koch JC , Tatenhorst L, Roser AE, Saal KA, Tönges L, Lingor P. ROCK inhibition in models of neurodegeneration and its potential for clinical translation. Pharmacol Ther. 2018;189:1–21. 29621594 10.1016/j.pharmthera.2018.03.008

[191] Kataura T , Tashiro E, Nishikawa S, et al A chemical genomics-aggrephagy integrated method studying functional analysis of autophagy inducers. Autophagy. 2021;17(8):1856–1872. 32762399 10.1080/15548627.2020.1794590 PMC8386610

[192] Ralph GS , Radcliffe PA, Day DM, et al Silencing mutant SOD1 using RNAi protects against neurodegeneration and extends survival in an ALS model. Nat Med. 2005;11(4):429–433. 15768029 10.1038/nm1205

[193] Raoul C , Abbas-Terki T, Bensadoun JC, et al Lentiviral-mediated silencing of SOD1 through RNA interference retards disease onset and progression in a mouse model of ALS. Nat Med. 2005;11(4):423–428. 15768028 10.1038/nm1207

[194] Bravo-Hernandez M , Tadokoro T, Navarro MR, et al Spinal subpial delivery of AAV9 enables widespread gene silencing and blocks motoneuron degeneration in ALS. Nat Med. 2020;26(1):118–130. 31873312 10.1038/s41591-019-0674-1 PMC8171115

[195] Miller TM , Pestronk A, David W, et al An antisense oligonucleotide against SOD1 delivered intrathecally for patients with SOD1 familial amyotrophic lateral sclerosis: A phase 1, randomised, first-in-man study. Lancet Neurol. 2013;12(5):435–442. 23541756 10.1016/S1474-4422(13)70061-9 PMC3712285

[196] Miller T , Cudkowicz M, Shaw PJ, et al Phase 1–2 trial of antisense oligonucleotide tofersen for SOD1 ALS. N Engl J Med. 2020;383(2):109–119. 32640130 10.1056/NEJMoa2003715

